# Nectin-4 and p95-ErbB2 cooperatively regulate Hippo signaling-dependent *SOX2* gene expression, enhancing anchorage-independent T47D cell proliferation

**DOI:** 10.1038/s41598-021-86437-2

**Published:** 2021-04-01

**Authors:** Shin Kedashiro, Takeshi Kameyama, Kiyohito Mizutani, Yoshimi Takai

**Affiliations:** Division of Pathogenetic Signaling, Department of Biochemistry and Molecular Biology, Kobe University Graduate School of Medicine, 1-5-6 Minatojima-minamimachi, Chuo-ku, Kobe, Hyogo 650-0047 Japan

**Keywords:** Biochemistry, Oncogenes

## Abstract

Nectin-4, upregulated in various cancer cells, *cis*-interacts with ErbB2 and its trastuzumab-resistant splice variants, p95-ErbB2 and ErbB2∆Ex16, enhancing DNA synthesis through the PI3K-AKT signaling in human breast cancer T47D cells in an adherent culture. We found here that nectin-4 and p95-ErbB2, but not nectin-4 and either ErbB2 or ErbB2∆Ex16, cooperatively enhanced *SOX2* gene expression and cell proliferation in a suspension culture. This enhancement of T47D cell proliferation in a suspension culture by nectin-4 and p95-ErbB2 was dependent on the *SOX2* gene expression. In T47D cells, nectin-4 and any one of p95-ErbB2, ErbB2, or ErbB2∆Ex16 cooperatively activated the PI3K-AKT signaling, known to induce the *SOX2* gene expression, to similar extents. However, only a combination of nectin-4 and p95-ErbB2, but not that of nectin-4 and either ErbB2 or ErbB2∆Ex16, cooperatively enhanced the *SOX2* gene expression. Detailed studies revealed that only nectin-4 and p95-ErbB2 cooperatively activated the Hippo signaling. YAP inhibited the *SOX2* gene expression in this cell line and thus the MST1/2-LATS1/2 signaling-mediated YAP inactivation increased the *SOX2* gene expression. These results indicate that only the combination of nectin-4 and p95-ErbB2, but not that of nectin-4 and either ErbB2 or ErbB2∆Ex16, cooperatively regulates the Hippo signaling-dependent *SOX2* gene expression, enhancing anchorage-independent T47D cell proliferation.

## Introduction

ErbB2 is a cell surface receptor that regulates various cellular events, including cell proliferation and migration via its tyrosine kinase activity^1–5^. ErbB2 belongs to the epidermal growth factor (EGF) receptor family, which consists of four members, ErbB1, ErbB2, ErbB3, and ErbB4. They are also known as HER1 (commonly called EGF receptor), HER2, HER3, and HER4, respectively^1–5^. ErbB2 plays roles in cell proliferation, survival, and differentiation in vitro and in vivo, and its upregulation by the gene amplification causes many types of cancers, including breast and gastric cancers^1–5^. Thus, ErbB2 serves as an oncogenic protein inducing tumorigenesis, invasion, and metastasis. In addition, the splice variants of ErbB2, p95-ErbB2 and ErbB2∆Ex16, are also expressed in some cases of breast cancers^6–17^. Trastuzumab (also known as Herceptin) is a monoclonal antibody (mAb) that targets ErbB2 and is clinically used for ErbB2-upregulated breast cancers as an antibody drug^18^. However, p95-ErbB2 and ErbB2∆Ex16 confer resistance to trastuzumab although trastuzumab-resistance to ErbB2∆Ex16 is still controversial^19,20^. ErbB2-upregulated breast cancers in all breast cancer cases are approximately 30%^21,22^. p95-ErbB2 is expressed in approximately 30% of the ErbB2-upregulated breast cancers, and that of ErbB2∆Ex16 is approximately 90%^11,23,24^. Therapeutic drugs for trastuzumab-resistant breast cancers expressing such variants have not been developed.

Nectin-4 is a cell adhesion molecule (CAM) that was first discovered by Lopez’s group^25^. Nectin-4 belongs to the nectin and nectin-like molecule (Necl) superfamily that comprises two families, the nectin family with four members (nectin-1, -2, -3, and -4) and the Necl family with five members (Necl-1, -2, -3, -4, and -5)^26–28^. Nectins and Necls homophilicaly or heterophilicaly *trans*-interact with each other and further *cis*-interact with many cell surface membrane receptors, including growth factor receptors, hormone receptors, and integrins, eventually regulating various cell functions, including cell proliferation, survival, migration, and differentiation, in addition to cell adhesion^29–33^.

Nectin-4 is upregulated in many types of cancers, such as breast, pancreatic, lung, gallbladder, ovarian, gastric, and bladder cancers^34–40^. Nectin-4 activates the PI3K-AKT signaling through the Rac small G protein signaling and the Wnt-β-catenin signaling for cancer cell proliferation and metastasis^36,37,41,42^. Moreover, nectin-4 promotes anchorage-independent cell survival in soft agar through the integrin β4-SHP2-c-Src signaling, which is activated by the *cis*-interaction of nectin-4 with integrin β4 and the *trans*-interaction of nectin-4 with nectin-1^41^. Nectin-4 further inhibits ferroptosis of epithelial and cancer cells by clustering each cell to survive under a matrix-detached condition^43^. These observations provide the concept that nectin-4 is an ideal target as a diagnostic marker and an anti-cancer drug. Indeed, Ab-drug conjugate enfortumab vedotin, a fully humanized mAb targeting nectin-4 linked to a microtubule-disrupting agent, has been approved for patients with advanced bladder cancer^44^.

We previously showed using human breast cancer T47D and SUM190-PT cells that nectin-4 *cis*-interacts with ErbB2 and enhances its homo-dimerization and activation in a novel mechanism, activating the PI3K-AKT signaling and enhancing DNA synthesis^33^. We further showed that nectin-4 also *cis*-interacts with the trastuzumab-resistant splice variants of ErbB2, p95-ErbB2 and ErbB2∆Ex16, activating the PI3K-AKT signaling and enhancing DNA synthesis^33^. In addition, nectin-4 activates the p95-ErbB2-induced JAK-STAT signaling, but not the ErbB2- or ErbB2∆Ex16-induced JAK-STAT signaling^33^.

We showed here using the T47D cells stably expressing both nectin-4 and any one of ErbB2, p95-ErbB2, or ErbB2∆Ex16 that only the combination of nectin-4 and p95-ErbB2, but not that of nectin-4 and eihter ErbB2 or ErbB2∆Ex16, cooperatively enhanced the *SOX2* gene expression and cell proliferation in a suspension culture. The enhancement of T47D cell proliferation in a suspension culture by nectin-4 and p95-ErbB2 was dependent on the *SOX2* gene expression. SOX2 is an essential transcription factor for maintaining self-renewal of undifferentiated embryonic and tissue stem cells, thus regulating embryogenesis and normal development^45,46^. SOX2 is also expressed in many types of cancer cells and regulates their proliferation, survival, differentiation, and tolerance to anti-cancer drugs^45–49^. The *SOX2* gene expression is induced by many signaling pathways including the PI3K-AKT signaling^45–49^. Detailed studies revealed here that nectin-4 and any one of p95-ErbB2, ErbB2, or ErbB2∆Ex16 cooperatively activated the PI3K-AKT signaling to similar extents^33^, but that only the combination of nectin-4 and p95-ErbB2, but not that of nectin-4 and either ErbB2 or ErbB2∆Ex16, cooperatively activated the Hippo signaling to regulate the *SOX2* gene expression. In this signaling pathway, MST1/2 phosphorylates and activates LATS1/2, which in turn phosphorylates YAP, keeping the phosphorylated YAP in the cytoplasm, thus inhibiting its activity^50^. YAP is a transcriptional co-activator that enhances cell proliferation and inhibits cell death by its association with the TEAD family of transcription factors^50^. YAP enhances or decreases the *SOX2* gene expression depending on cell types^49,51–54^, and we showed here that YAP inhibited the *SOX2* gene expression in T47D cells. Thus, nectin-4 and p95-ErbB2 activated the MST1/2-LATS1/2 signaling to suppress the inhibitory role of YAP in the PI3K-AKT signaling-mediated *SOX2* gene expression, eventually enhancing T47D cell proliferation through upregulated SOX2 in a serum-free suspension culture. We showed here the roles of nectin-4 and p95-ErbB2 in T47D cell proliferation in a suspension culture through the Hippo signaling-dependent *SOX2* gene expression.

## Results

### Enhancement of T47D cell proliferation by only the combinations of nectin-4 and p95-ErbB2, but not by that of nectin-4 and either ErbB2 or ErbB2∆Ex16, in a suspension culture

**Figure 1:**
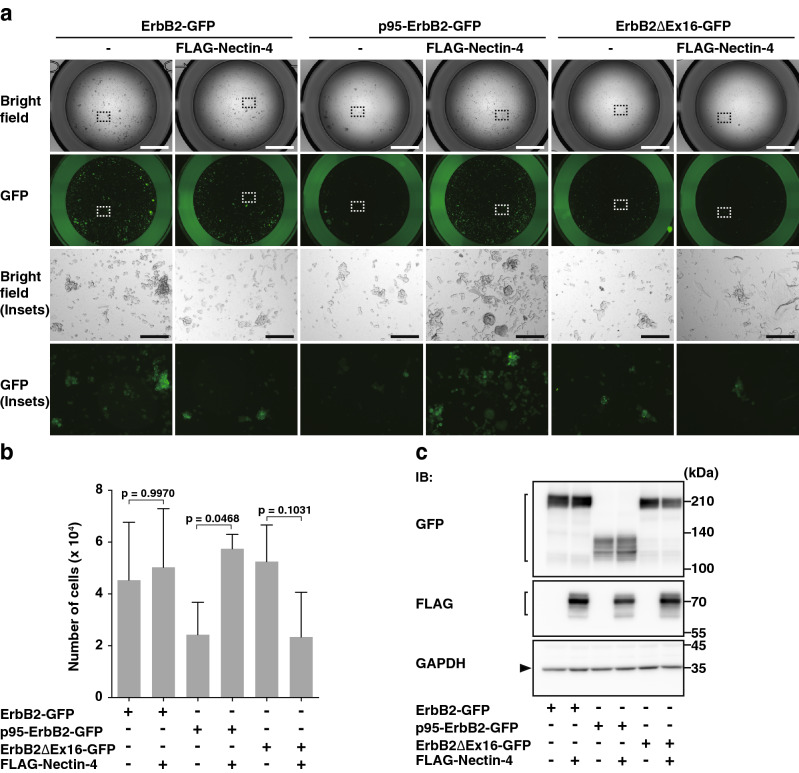
Enhancement of T47D cell proliferation by only the combination of nectin-4 and p95-ErbB2, but not by that of nectin-4 and either ErbB2 or ErbB2∆Ex16, in a suspension culture. (**a**,**b**) Enhancement of T47D cell proliferation by only the combination of nectin-4 and p95-ErbB2, but not by that of nectin-4 and either ErbB2 or ErbB2∆Ex16, in a suspension culture. The T47D cells stably expressing GFP-tagged ErbB2 or each of its splice variants with or without FLAG-tagged nectin-4 (FLAG-Nectin-4) were detached using Accutase. The cells were collected, their numbers were counted, and the same numbers of the cells were seeded in the serum-free medium with supplements on ultra-low attachment 6 well dish. After the incubation for 28 days, the cells were collected and transferred to ultra-low attachment 96 well dish for image acquisition. Then, the cells were re-collected and subjected to quantitative analysis by cell counting as shown in (**b**). The displayed images were acquired using a BZ-X710 microscope and its software BZ-X Analyzer (https://www.keyence.co.jp/products/microscope/fluorescence-microscope/bz-x700/models/bz-x710/) with BZ-H3A Advanced Application software (https://www.keyence.co.jp/products/microscope/fluorescence-microscope/bz-x700/models/bz-h3a/) for image connection. (**c**) Amounts of nectin-4, ErbB2, and its splice variant proteins. The T47D cells stably expressing GFP-tagged ErbB2 or each of its splice variants with or without FLAG-Nectin-4 were cultured for 72 h in an adherent culture. The cells were subjected to Western blotting using the indicated antibodies (Abs). All the cell lines used in the experiments were the bulk of collected cells and not singly picked-up clones. Arrowhead and square brackets indicate each of the proteins. The displayed blots were cropped, and the full-length blots are shown in Supplementary Figure S3. Bars indicate the means ± S.D. of five independent experiments and the actual P values for each test are shown. Scale bars 2000 μm or 200 μm (insets). IB, immunoblotting. Representative results (images) from three independent experiments were shown.

Anchorage-independent cell proliferation is one of the characteristics of cancer cells^55^. We therefore examined the effect of nectin-4 and any one of ErbB2, p95-ErbB2, or ErbB2∆Ex16 on T47D cell proliferation in a suspension culture. The reason why human breast cancer T47D cells were used was that they endogenously express both nectin-4 and ErbB2^33^. We first established the T47D cells stably expressing ErbB2 alone (ErbB2-T47D cells), both FLAG-nectin-4 and ErbB2 (nectin-4-ErbB2-T47D cells), p95-ErbB2 alone (p95-ErbB2-T47D cells), both FLAG-nectin-4 and p95-ErbB2 (nectin-4-p95-ErbB2-T47D cells), ErbB2∆Ex16 alone (ErbB2∆Ex16-T47D cells), and both FLAG-nectin-4 and ErbB2∆Ex16 (nectin-4-ErbB2∆Ex16-T47D cells). When these cell lines were cultured in suspension for 28 days, all of them proliferated and formed various sizes of aggregates. To visually evaluate each cell line proliferation in a suspension culture, each cell line was collected and transferred to smaller dishes for the image acquisition. After the image acquisition, the cells were then re-collected to calculate a cell number by DNA-amount-based cell-counting method. The number of nectin-4-p95-ErbB2-T47D cells was larger than that of p95-ErbB2-T47D cells but was similar to those of nectin-4-ErbB2-T47D and ErbB2ΔEx16-T47D cells (Fig. 1a,b). The number of ErbB2-T47D cells was similar to that of nectin-4-ErbB2-T47D cells, and the number of ErbB2ΔEx16-T47D cells was larger than that of nectin-4-ErbB2ΔEx16-T47D cells, but this result for ErbB2ΔEx16-T47D and nectin-4-ErbB2ΔEx16-T47D cells was not statistically significant. These results indicate that only the combination of nectin-4 and p95-ErbB2, but not that of nectin-4 and either ErbB2 or ErbB2∆Ex16, cooperatively enhances T47D cell proliferation in a suspension culture. Although the mean number of ErbB2ΔEx16-T47D cells was slightly larger than those of ErbB2-T47D and nectin-4-ErbB2-T47D cells, the numbers of ErbB2-T47D cells and nectin-4-ErbB2-T47D cells were occasionally larger than that of ErbB2ΔEx16-T47D cells in three experiments. The numbers of ErbB2-T47D and nectin-4-ErbB2-T47D cells were larger than that of p95-ErbB2-T47D cells. This was due to the larger amounts of the ErbB2 protein expressed in ErbB2-T47D and nectin-4-ErbB2-T47D cells than those of the p95-ErbB2 protein expressed in p95-ErbB2-T47D and nectin-4-p95-ErbB2-T47D cells and those of the ErbB2∆Ex16 protein expressed in ErbB2∆Ex16-T47D and nectin-4-ErbB2∆Ex16-T47D cells (Fig. 1c). The amounts of the p95-ErbB2 and ErbB2∆Ex16 proteins were similar in p95-ErbB2-T47D, nectin-4-p95-ErbB2-T47D, ErbB2∆Ex16-T47D, and nectin-4-ErbB2∆Ex16-T47D cells.

### PI3K-AKT signaling- and Ras-Raf-MEK-ERK signaling-dependent, but not JAK-STAT signaling-dependent, enhancement of T47D cell proliferation by nectin-4 and p95-ErbB2 in a suspension culture

**Figure 2:**
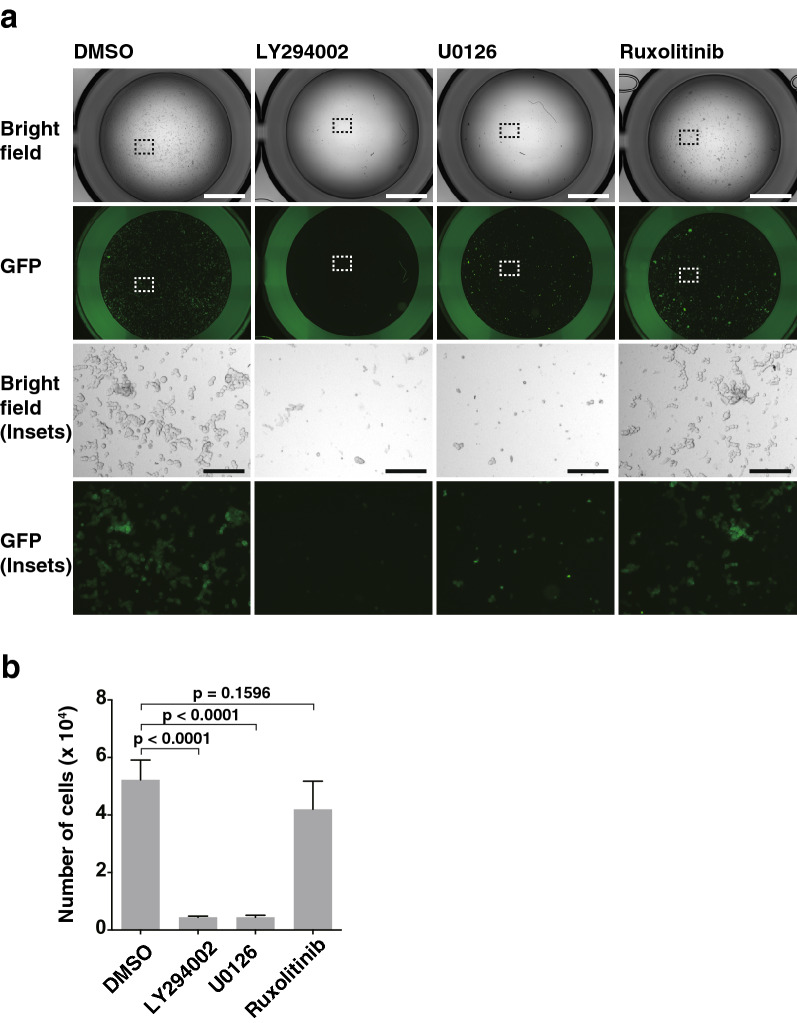
PI3K-AKT signaling- and the Ras-Raf-MEK-ERK signaling-dependent, but not the JAK-STAT signaling-dependent, enhancement of T47D cell proliferation by nectin-4 and p95-ErbB2 in a suspension culture. (**a**,**b**) The PI3K-AKT signaling- and the Ras-Raf-MEK-ERK signaling-dependent, but not the JAK-STAT signaling-dependent, enhancement of T47D cell proliferation by nectin-4 and p95-ErbB2 in a suspension culture. The T47D cells stably expressing GFP-tagged p95-ErbB2 (p95-ErbB2-GFP) with FLAG-tagged nectin-4 (FLAG-Nectin-4) were detached using Accutase. The cells were collected, their numbers were counted, and the same numbers of the cells were seeded in the serum-free medium with supplements on ultra-low attachment 6 well dish. After the incubation with the PI3K inhibitor LY294002 at 50 μM, the MEK inhibitor U0126 at 10 μM, or the JAK1/2 inhibitor ruxolitinib at 1 μM for 28 days, the cells were collected and transferred to ultra-low attachment 96 well dish for image acquisition. Then, the cells were re-collected and subjected to quantitative analysis by cell counting as shown in (**b**). The displayed images were acquired using a BZ-X710 microscope and its software BZ-X Analyzer (https://www.keyence.co.jp/products/microscope/fluorescence-microscope/bz-x700/models/bz-x710/) with BZ-H3A Advanced Application software (https://www.keyence.co.jp/products/microscope/fluorescence-microscope/bz-x700/models/bz-h3a/) for image connection. The T47D cells stably expressing p95-ErbB2-GFP with FLAG-Nectin-4 used in the experiments were the bulk of collected cells and not singly picked-up clones. Bars indicate the means ± S.D. of three independent experiments and the actual P values for each test are shown. Scale bars 2000 μm or 200 μm (insets). Representative results (images) from three independent experiments were shown.

We previously showed that only the combination of nectin-4 and p95-ErbB2, but not that of nectin-4 and either ErbB2 or ErbB2∆Ex16, activates the JAK-STAT signaling in addition to the PI3K-AKT signaling in T47D cells in an adherent culture^33^. We therefore examined whether the JAK-STAT signaling is involved in the enhancement of T47D cell proliferation by nectin-4 and p95-ErbB2 in a suspension culture. The enhancement of T47D cell proliferation by nectin-4 and p95-ErbB2 in a suspension culture was inhibited by the PI3K inhibitor LY294002 and the MEK inhibitor U0126, but not the JAK1/2 inhibitor ruxolitinib (Fig. 2a,b). These results indicate that the PI3K-AKT signaling and the Ras-Raf-MEK-ERK signaling, but not the JAK-STAT signaling, were required for the enhancement of T47D cell proliferation by nectin-4 and p95-ErbB2 in a suspension culture.

### PI3K-AKT signaling-dependent increase in the amount of the SOX2 protein by only the combination of nectin-4 and p95-ErbB2, but not by that of nectin-4 and either ErbB2 or ErbB2∆Ex16, in T47D cells in an adherent culture

**Figure 3:**
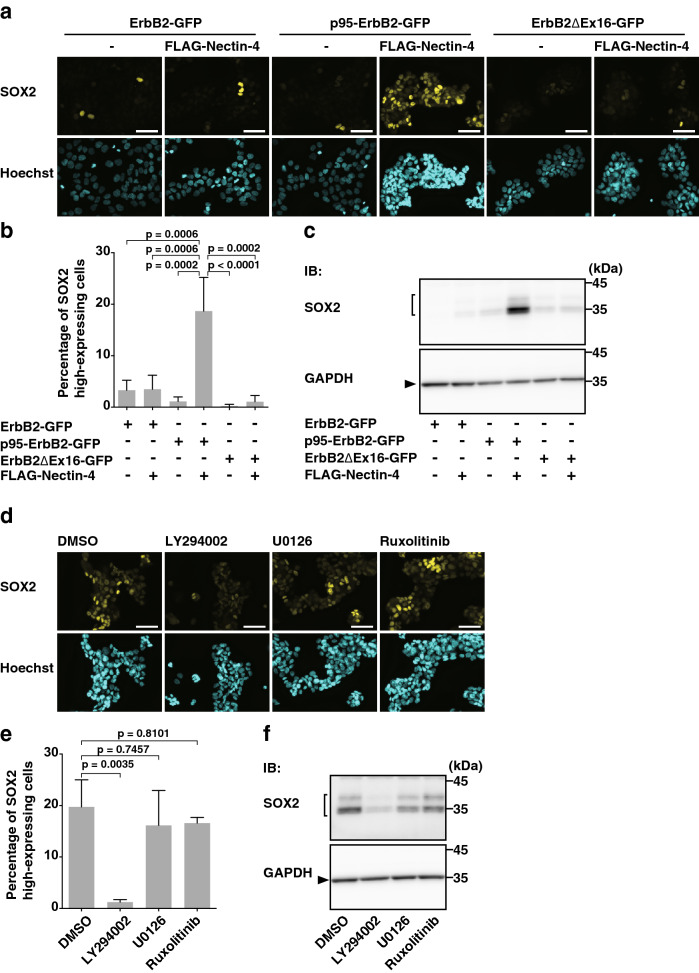
PI3K-AKT signaling-dependent increase in the amount of the SOX2 protein by only the combination of nectin-4 and p95-ErbB2, but not by that of nectin-4 and either ErbB2 or ErbB2∆Ex16, in T47D cells cultured in an adherent culture. (**a**–**c**) Increase in the amount of the SOX2 protein by only the combination of nectin-4 and p95-ErbB2, but not by that of nectin-4 and either ErbB2 or ErbB2ΔEx16, in T47D cells in an adherent culture. The T47D cells stably expressing GFP-tagged ErbB2 or each of its splice variants with or without FLAG-tagged nectin-4 (FLAG-Nectin-4) were cultured for 72 h in an adherent culture. The cells were fixed and stained with the indicated Ab and Hoechst33342 as shown in (**a**). The number of the cells that prominently express SOX2 was counted by fluorescence microscopy as shown in (**b**). The T47D cells stably expressing GFP-tagged ErbB2 or each of its splice variants with or without FLAG-Nectin-4 were cultured for 72 h in an adherent culture. The cells were subjected to Western blotting using the indicated Abs as shown in (**c**). Arrowhead and square brackets indicate each of the proteins. The displayed blots were cropped, and the full-length blots are shown in Supplementary Figure S4a. (**d**–**f**) Requirement of the PI3K-AKT signaling-, but not the Ras-Raf-MEK-ERK signaling-dependent or the JAK-STAT signaling-dependent increase in the amount of the SOX2 protein by nectin-4 and p95-ErbB2 in T47D cells in an adherent culture. The T47D cells stably expressing GFP-tagged p95-ErbB2 (p95-ErbB2-GFP) with FLAG-Nectin-4 were cultured for 48 h. The cells were then treated with the PI3K inhibitor LY294002 at 50 μM, the MEK inhibitor U0126 at 10 μM, or the JAK1/2 inhibitor ruxolitinib at 1 μM for 24 h. After the incubation, the assays were carried out as in (**a**–**c**). The displayed images were acquired using a BZ-X710 microscope and its software BZ-X Analyzer (https://www.keyence.co.jp/products/microscope/fluorescence-microscope/bz-x700/models/bz-x710/) and processed using ImageJ version 1.48v 32-bit software (https://imagej.nih.gov/ij/) for color changes of the images. The displayed blots were cropped, and the full-length blots are shown in Supplementary Figure S4b. All the cell lines used in the experiments were the bulk of collected cells and not singly picked-up clones. Bars indicate the means ± S.D. of three independent experiments and the actual P values for each test are shown in each figure. Scale bars 50 μm. IB, immunoblotting. Representative results (images) from three independent experiments were shown.

SOX2 is expressed in cancer cells and regulates their proliferation, migration, invasion, and metastasis^45–49^. We therefore examined whether nectin-4 and p95-ErbB2 cooperatively increase the amount of the SOX2 protein in T47D cells in an adherent culture. The amount of the SOX2 protein expressed in nectin-4-p95-ErbB2-T47D cells was higher than that expressed in other cell lines (Fig. 3a–c). This increase in the SOX2 protein by nectin-4 and p95-ErbB2 in nectin-4-p95-ErbB2-T47D cells was inhibited by LY294002, but not by U0126 or ruxolitinib (Fig. 3d–f). These results indicate that only the combination of nectin-4 and p95-ErbB2, but not that of nectin-4 and either ErbB2 or ErbB2∆Ex16, cooperatively increases the amount of the SOX2 protein and that this increase in the SOX2 protein is dependent on the PI3K-AKT signaling, but not on the Ras-Raf-MEK-ERK signaling or the JAK-STAT signaling.

### SOX2-dependent enhancement of T47D cell proliferation by nectin-4 and p95-ErbB2 in a suspension culture

**Figure 4:**
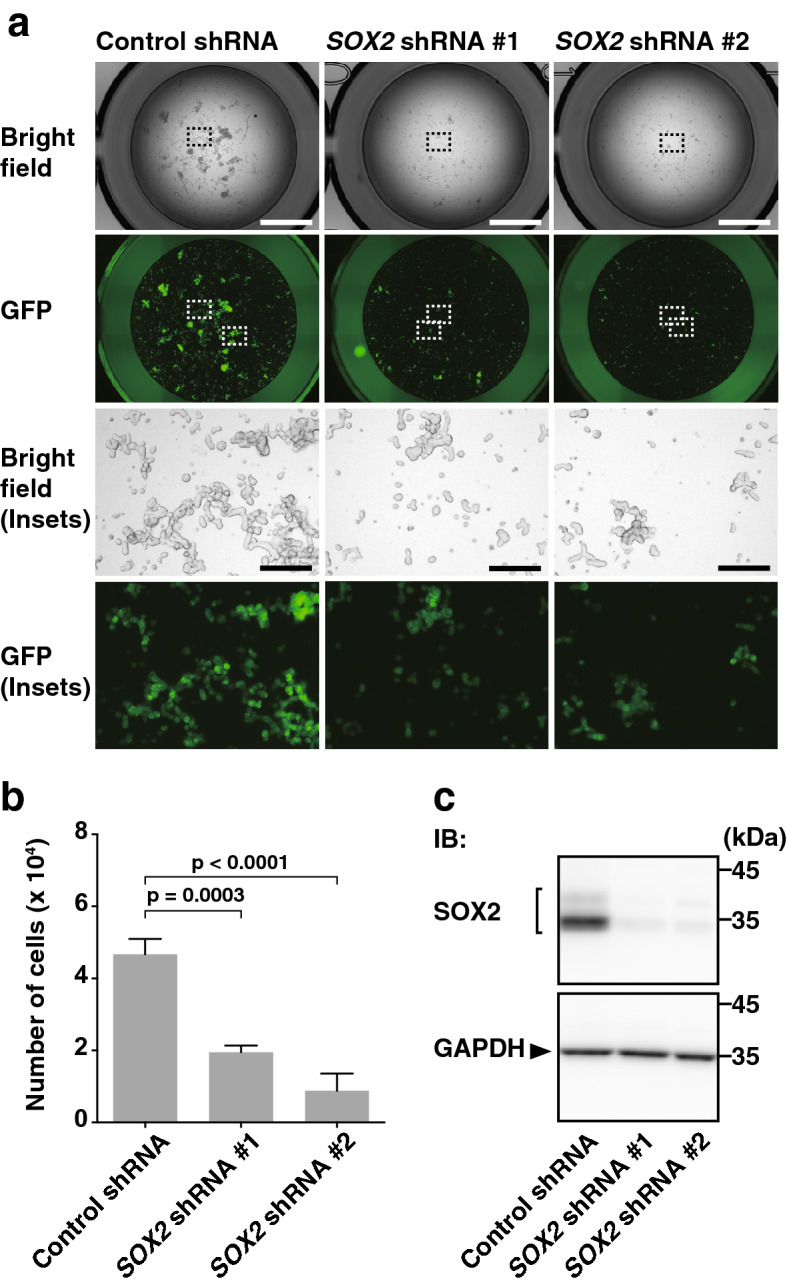
SOX2-dependent enhancement of T47D cell proliferation by nectin-4 and p95-ErbB2 in a suspension culture. (**a**,**b**) Requirement of SOX2 for the enhancement of T47D cell proliferation by nectin-4 and p95-ErbB2 in a suspension culture. The T47D cells stably expressing GFP-tagged p95-ErbB2 (p95-ErbB2-GFP) with FLAG-tagged nectin-4 (FLAG-Nectin-4) that also stably express control shRNA or *SOX2* shRNAs were detached using Accutase. The cells were collected, their numbers were counted, and the same numbers of the cells were seeded in the serum-free medium with supplements on ultra-low attachment 6 well dish. After the incubation for 28 days, the cells were collected and transferred to ultra-low attachment 96 well dish for image acquisition. Then, the cells were re-collected and subjected to quantitative analysis by cell counting as shown in (**b**). The displayed images were acquired using a BZ-X710 microscope and its software BZ-X Analyzer (https://www.keyence.co.jp/products/microscope/fluorescence-microscope/bz-x700/models/bz-x710/) with BZ-H3A Advanced Application software (https://www.keyence.co.jp/products/microscope/fluorescence-microscope/bz-x700/models/bz-h3a/) for image connection. (**c**) Decrease in the amount of the SOX2 protein by *SOX2* knockdown. The T47D cells stably expressing p95-ErbB2-GFP with FLAG-Nectin-4 that also stably express control shRNA or *SOX2* shRNAs were cultured for 72 h in an adherent culture. The cells were subjected to Western blotting using the indicated Abs. Arrowhead and square bracket indicate each of the proteins. The displayed blots were cropped, and the full-length blots are shown in Supplementary Figure S5. All the cell lines used in the experiments were the bulk of collected cells and not singly picked-up clones. Bars indicate the means ± S.D. of three independent experiments and the actual P values for each test are shown. Scale bars 2000 μm or 200 μm (insets). IB, immunoblotting. Representative results (images) from three independent experiments were shown.

We then examined by *SOX2* knockdown whether it is involved in the enhancement of　T47D cell proliferation by nectin-4 and p95-ErbB2 in a suspension culture. The amount of the SOX2 protein was markedly reduced by the shRNAs against *SOX2* in nectin-4-p95-ErbB2-T47D cells (Fig. 4c). In these *SOX2*-knockdown cells, the enhancement of T47D cell proliferation by nectin-4 and p95-ErbB2 in a suspension culture was markedly reduced, compared to that in control nectin-4-p95-ErbB2-T47D cells (Fig. 4a,b). These results indicate that the enhancement of T47D cell proliferation by nectin-4 and p95-ErbB2 in a suspension culture is dependent on SOX2.

### Shh, Wnt, FGF, and TGF-β receptor signaling-independent increase in the amount of the SOX2 protein by nectin-4 and p95-ErbB2 in T47D cells in an adherent culture

We previously showed that not only the combination of nectin-4 and p95-ErbB2 but also that of nectin-4 and either ErbB2 or ErbB2∆Ex16 activates the PI3K-AKT signaling to similar extents^33^. However, only the combination of nectin-4 and p95-ErbB2, but not that of nectin-4 and either ErbB2 or ErbB2∆Ex16, cooperatively increased the amount of the SOX2 protein in T47D cells as described above. Therefore, another signaling was expected to be involved in the increase in the amount of the SOX2 protein by nectin-4 and p95-ErbB2 in addition to the PI3K-AKT signaling. We searched for this another signaling. The *SOX2* gene expression is induced through at least four signaling pathways downstream of the Sonic hedgehog (Shh), Wnt, fibroblast growth factor (FGF), and transforming growth factor-β (TGF-β) receptors: Shh binds to its receptor Patched, activating the G-protein-coupled receptor Smoothened, eventually inducing the GLI1/2/3 direct activation and its translocation into the nucleus to induce the *SOX2* gene expression^56,57^; Wnt binds to its receptor Frizzled associated with its coreceptors LRP5/LRP6 to induce the GLI1/2/3 activation through the Dsh/GSK-3β/Axin/APC-β-catenin signaling^58^; FGF binds to its receptor and induces the *SOX2* gene expression through the PI3K-AKT-mTOR signaling and the Ras-Raf-MEK-ERK signaling^59^, and TGF-β binds to its receptor and induces the *SOX2* gene expression through the Smad2/3 signaling^60–62^. Among these signaling pathways, we first neglected the involvement of the FGF receptor signaling in the increase in the amount of the SOX2 protein by nectin-4 and p95-ErbB2, because the increase in the amount of the SOX2 protein was not inhibited by the MEK inhibitor U0126 in nectin-4-p95-ErbB2-T47D cells (Fig. 3d–f), and we previously showed that the PI3K-AKT signaling is enhanced not only by the combination of nectin-4 and p95-ErbB2 but also by that of nectin-4 and either ErbB2 or ErbB2∆Ex16 to similar extents^33^. These results suggest that the Ras-Raf-MEK-ERK signaling is not required for the increase in the amount of the SOX2 protein in these cells, and it is less likely that the increase in the SOX2 protein is induced only by the PI3K-AKT signaling in nectin-4-p95-ErbB2-T47D cells, but not in nectin-4-ErbB2-T47D cells or nectin-4-ErbB2∆Ex16-T47D cells. We therefore examined whether nectin-4 and p95-ErbB2 cooperatively enhance the three remaining signaling pathways. However, nectin-4 and any one of ErbB2, p95-ErbB2, or ErbB2∆Ex16 did not cooperatively enhance the GLI1 and GLI3 activation, the β-catenin activation, or the Smad2/3 activation in T47D cells (Supplementary Fig. S1). These results indicate that nectin-4 and p95-ErbB2 increase the amount of the SOX2 protein in a manner independent of the four signaling pathways downstream of the Shh, Wnt, FGF, and TGF-β receptors and raised the possibility that another signaling pathway except these ones is involved in the increase in the amount of the SOX2 protein by nectin-4 and p95-ErbB in addition to the PI3K-AKT signaling.

### Hippo signaling-dependent *SOX2* gene expression by nectin-4 and p95-ErbB2 in T47D cells in an adherent culture

**Figure 5:**
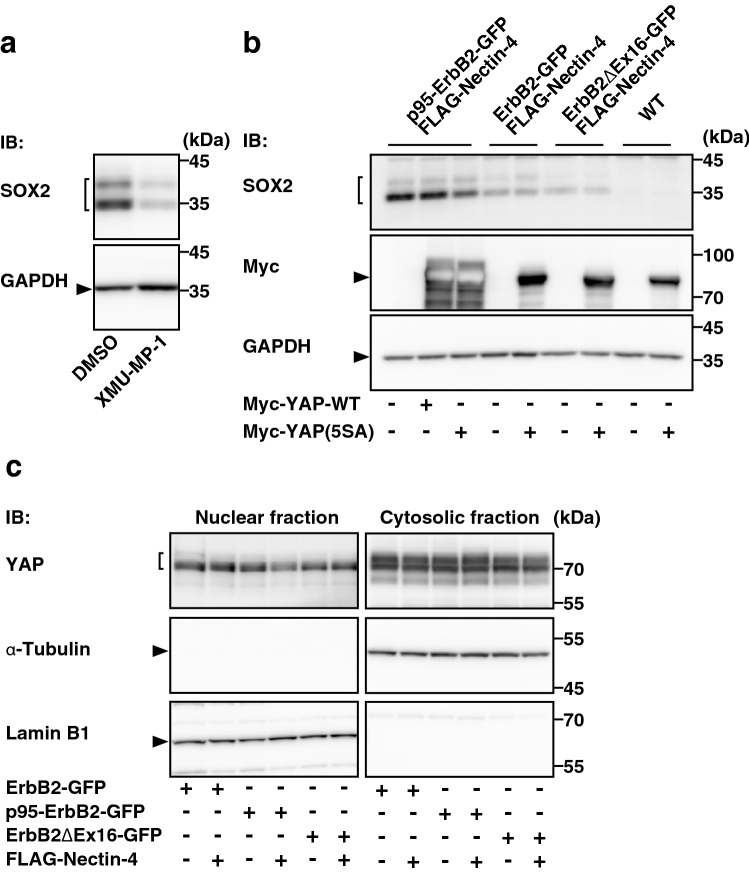
Hippo signaling-dependent *SOX2* gene expression by nectin-4 and p95-ErbB2 in T47D cells in an adherent culture. (**a**) Requirement of the MST1/2 activation for the increase in the amount of the SOX2 protein by nectin-4 and p95-ErbB2 in T47D cells in an adherent culture. The T47D cells stably expressing GFP-tagged p95-ErbB2 (p95-ErbB2-GFP) with FLAG-tagged nectin-4 (FLAG-Nectin-4) were cultured for 48 h. The cells were then treated with the MST1/2 inhibitor XMU-MP-1 at 1 μM for 24 h. The cells were subjected to Western blotting using the indicated Abs. (**b**) Decrease by a constitutively active YAP in the amount of the SOX2 protein increased by nectin-4 and p95-ErbB2 in T47D cells in an adherent culture. The T47D cells stably expressing GFP-tagged ErbB2 or each of its splice variants with FLAG-Nectin-4 were transfected with Myc-tagged YAP wild-type (Myc-YAP-WT) or Myc-tagged YAP(5SA) mutant (Myc-YAP(5SA)). The cells were then cultured for 72 h in an adherent culture. The cells were subjected to Western blotting using the indicated Abs. (**c**) Decrease in the amount of the YAP protein in the nuclear fraction by nectin-4 and p95-ErbB2 in T47D cells in an adherent culture. The nuclear and cytosolic fractions were prepared from the T47D cells stably expressing GFP-tagged ErbB2 or each of its splice variants with or without FLAG-Nectin-4. Each fraction was subjected to SDS-PAGE followed by Western blotting using the indicated Abs. All the cell lines used in the experiments were the bulk of collected cells and not singly picked-up clones. Arrowheads and square brackets indicate each of the proteins. The displayed blots were cropped, and the full-length blots are shown in Supplementary Figure S6a–c. IB, immunoblotting. Representative results (images) from three independent experiments were shown.

*SOX2* gene expression is regulated through the Hippo signaling in addition to the above-mentioned four signaling pathways^49,51–54^. The Hippo signaling regulates diverse events, including cell proliferation and apoptosis^50^. The MST1/2 inhibitor XMU-MP-1 inhibited the increase in the amount of the SOX2 protein by nectin-4 and p95-ErbB2 in T47D cells in an adherent culture (Fig. 5a), indicating that the increase in the amount of the SOX2 protein is dependent on the inhibition of the phosphorylated YAP translocation from the cytoplasm to the nucleus. On the other hand, exogenous expression of a non-phosphorylated form of Myc-tagged YAP (Myc-YAP(5SA)), which acts as a constitutively active mutant^63^, in nectin-4-p95-ErbB2-T47D cells decreased the increased amount of the SOX2 protein (Fig. 5b), although exogenous expression of Myc-YAP(5SA) in nectin-4-ErbB2-T47D cells, nectin-4-ErbB2∆Ex16-T47D cells, or wild-type T47D cells did not affect the amount of the SOX2 protein (Fig. 5b). To confirm whether YAP is indeed inhibited in nectin-4-p95-ErbB2-T47D cells, the nuclear and cytosolic fractions were prepared from ErbB2-T47D, nectin-4-ErbB2-T47D, p95-ErbB2-T47D, nectin-4-p95-ErbB2-T47D, ErbB2∆Ex16-T47D, and nectin-4-ErbB2∆Ex16-T47D cells, and the amount of the YAP protein in each fraction was measured by Western blotting with lamin B1 as a nuclear marker and α-tubulin as a cytosolic marker. The amount of the YAP protein in the nuclear fraction was lower in nectin-4-p95-ErbB2-T47D cells than in ErbB2-T47D, nectin-4-ErbB2-T47D, p95-ErbB2-T47D, ErbB2∆Ex16-T47D, and nectin-4-ErbB2∆Ex16-T47D cells (Fig. 5c). Collectively, these results indicate that nectin-4 and p95-ErbB2 cooperatively enhance the Hippo signaling-dependent *SOX2* gene expression in T47D cells in an adherent culture.

### YAP inactivation-dependent enhancement of T47D cell proliferation by nectin-4 and p95-ErbB2 in a suspension culture

**Figure 6:**
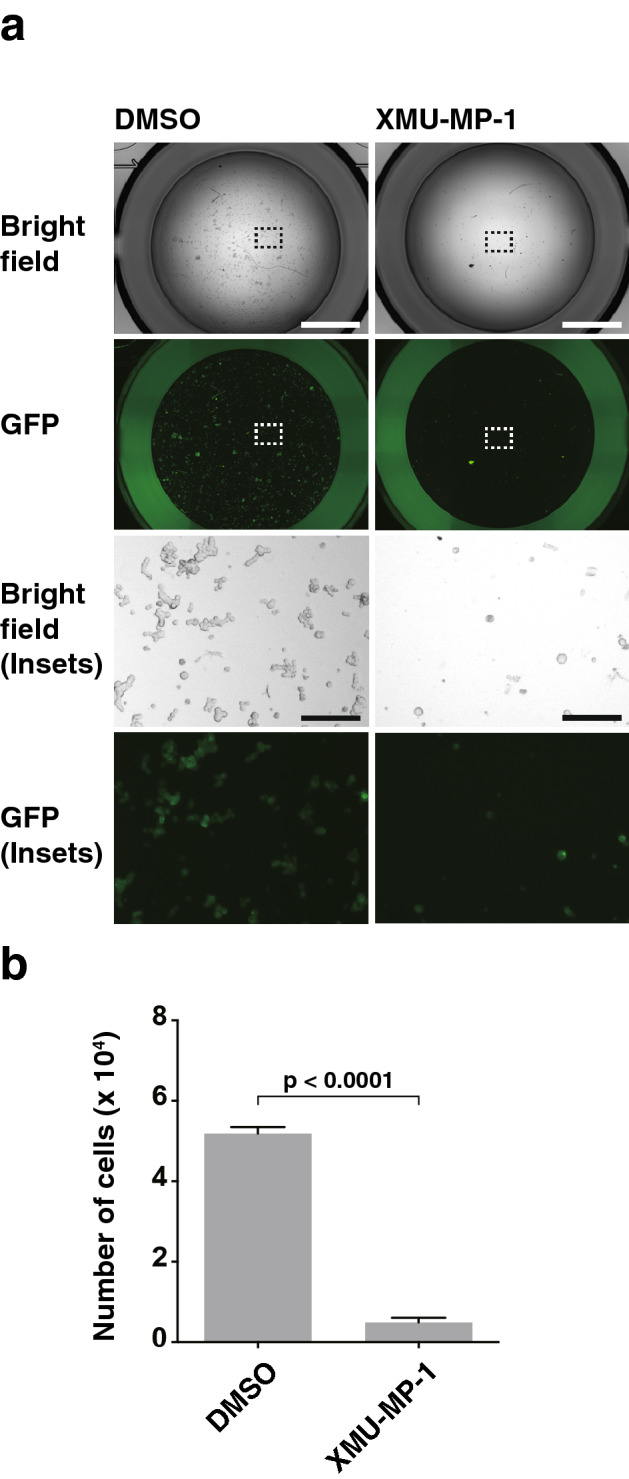
YAP inactivation-dependent enhancement of T47D cell proliferation by nectin-4 and p95-ErbB2 in a suspension culture. (**a**,**b**) Requirement of the MST1/2 activation for the enhancement of T47D cell proliferation in a suspension culture by nectin-4 and p95-ErbB2. The T47D cells stably expressing GFP-tagged p95-ErbB2 (p95-ErbB2-GFP) with FLAG-tagged nectin-4 (FLAG-Nectin-4) were detached using Accutase. The cells were collected, their numbers were counted, and the same numbers of the cells were seeded in serum-free medium with supplements on ultra-low attachment 6 well dish. After the incubation with the MST1/2 inhibitor XMU-MP-1 at 1 μM for 28 days, the cells were collected and transferred to ultra-low attachment 96 well dish for image acquisition. Then, the cells were re-collected and subjected to quantitative analysis by cell counting as shown in (**b**). The displayed images were acquired using a BZ-X710 microscope and its software BZ-X Analyzer (https://www.keyence.co.jp/products/microscope/fluorescence-microscope/bz-x700/models/bz-x710/) with BZ-H3A Advanced Application software (https://www.keyence.co.jp/products/microscope/fluorescence-microscope/bz-x700/models/bz-h3a/) for image connection. The T47D cells stably expressing p95-ErbB2-GFP with FLAG-Nectin-4 used in the experiments were the bulk of collected cells and not singly picked-up clones. Bars indicate the means ± S.D. of three independent experiments and the actual P value is shown in each figure. Scale bars 2000 μm or 200 μm (insets). Representative results (images) from three independent experiments were shown.

We then examined whether the enhancement of T47D cell proliferation by nectin-4 and p95-ErbB2 in a suspension culture is dependent on the Hippo signaling. The MST1/2 inhibitor XMU-MP-1 inhibited the enhancement of T47D cell proliferation by nectin-4 and p95-ErbB2 in a suspension culture (Fig. 6a,b). Collectively, these results indicate that the enhancement of T47D cell proliferation by nectin-4 and p95-ErbB2 in a suspension culture is dependent on the Hippo signaling.

### CD44, angiomotin, or merlin-independent costimulatory effects of nectin-4 and p95-ErbB2 in T47D cells in an adherent culture

We finally examined how nectin-4 and p95-ErbB2 cooperatively activate the Hippo signaling-dependent *SOX2* gene expression and enhance T47D cell proliferation in a suspension culture. CD44, angiomotin, and merlin are upstream regulators for the Hippo signaling and associated with cell adhesion^64–68^. The immunofluorescence signal for E-cadherin was not markedly different among the T47D cells stably expressing both nectin-4 and any one of ErbB2, p95-ErbB2, or ErbB2∆Ex16 (Supplementary Fig. S2a–c). Under these conditions, the CD44, angiomotin, or merlin expression was not markedly different among these types of cells (Supplementary Fig. S2d). These results indicate that nectin-4 and any one of p95-ErbB2, ErbB2, or ErbB2∆Ex16 do not affect the CD44, angiomotin, or merlin localization, suggesting that these molecules are not involved in the costimulatory effects of nectin-4 and p95-ErbB2 on the Hippo signaling-dependent *SOX2* gene expression and the T47D cell proliferation in a suspension culture, although their involvement in these effects was not completely excluded.

## Discussion

We previously showed that nectin-4 enhances DNA synthesis cooperatively with all ErbB2, p95-ErbB2, and ErbB2∆Ex16 in T47D cells in an adherent culture^33^. However, we found here that when T47D cells were cultured in suspension, only the combination of nectin-4 and p95-ErbB2, but not that of nectin-4 and either ErbB2 or ErbB2∆Ex16, cooperatively enhanced T47D cell proliferation. The tyrosine-phosphorylation of ErbB2 activates the PI3K-AKT signaling, the Ras-Raf-MEK-ERK signaling, and the JAK-STAT signaling^1–5,69–73^. We previously showed that nectin-4 *cis*-interacts with ErbB2 and induces the ErbB2-mediated DNA synthesis by mainly activating the ErbB2-mediated PI3K-AKT signaling, but not the Ras-Raf-MEK-ERK signaling or the JAK-STAT signaling, although the nectin-4-independent ErbB2-mediated Ras-Raf-MEK-ERK signaling, but not the JAK-STAT signaling, is involved in the ErbB2-mediated DNA synthesis^33^. We further showed that nectin-4 *cis*-interacts with not only ErbB2 but also p95-ErbB2 and ErbB2∆Ex16 and enhances the ErbB2-, p95-ErbB2-, and ErbB2∆Ex16-mediated DNA synthesis^33^. Nectin-4 enhances mainly the PI3K-AKT signaling and hardly the Ras-Raf-MEK-ERK signaling induced by ErbB2, p95-ErbB2, and ErbB2∆Ex16, but nectin-4 further enhances the JAK-STAT signaling induced by p95-ErbB2, but not by ErbB2 or ErbB2∆Ex16^33^. This nectin-4-enhanced PI3K-AKT signaling is involved in the nectin-4-enhanced DNA synthesis, although the nectin-4-independent ErbB2-, p95-ErbB2-, and ErbB2∆Ex16-mediated Ras-Raf-MEK-ERK signaling, but not the JAK-STAT signaling, is involved in the ErbB2-, p95-ErbB2-, and ErbB2∆Ex16-mediated DNA synthesis, respectively^33^. We showed here that the enhancement of T47D cell proliferation by nectin-4 and p95-ErbB2 in a suspension culture was dependent on the PI3K-AKT signaling and the Ras-Raf-MEK-ERK signaling, but not the JAK-STAT signaling. These results did not explain the specific costimulatory effect of nectin-4 and p95-ErbB2 on the T47D cell proliferation in a suspension culture, because nectin-4 and any one of ErbB2, p95-ErbB2, or ErbB2∆Ex16 cooperatively activate the PI3K-AKT signaling to similar extents and the JAK-STAT signaling activated by the costimulatory effect of only the combination of nectin-4 and p95-ErbB2, but not that of nectin-4 and either ErbB2 or ErbB2∆Ex16, was not involved in the costimulatory effect of nectin-4 and p95-ErbB2 on the T47D cell proliferation in a suspension culture.

We then showed here that only the combination of nectin-4 and p95-ErbB2, but not that of nectin-4 and either ErbB2 or ErbB2∆Ex16, cooperatively increased the amount of the SOX2 protein in T47D cells in an adherent culture. The increase in the amount of the SOX2 protein by nectin-4 and p95-ErbB2 was dependent on the PI3K-AKT signaling, but not the Ras-Raf-MEK-ERK signaling or the JAK-STAT signaling. The PI3K-AKT signaling both induces the *SOX2* gene expression and inhibits the proteolytic degradation of the SOX2 protein, thus increasing the amount of the SOX2 protein^48,74,75^. However, we previously showed that nectin-4 and any one of p95-ErbB2, ErbB2, or ErbB2∆Ex16 all cooperatively activate the PI3K-AKT signaling to similar extents^33^, but we showed here that only the combination of nectin-4 and p95-ErbB2, but not that of nectin-4 and either ErbB2 or ErbB2∆Ex16, cooperatively increased the amount of the SOX2 protein in T47D cells. These results raised the possibility that another signaling is involved in the increase in the amount of the SOX2 protein by nectin-4 and p95-ErbB2 in addition to the PI3K-AKT signaling.

**Figure 7:**
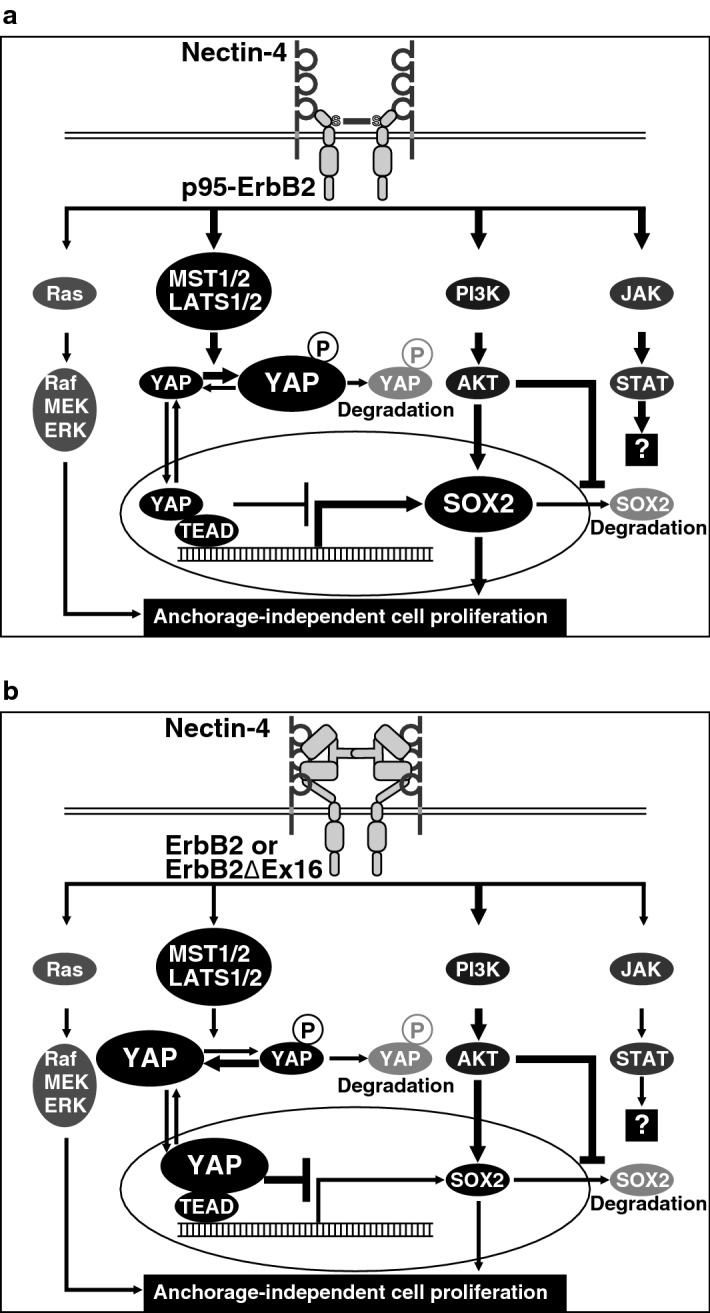
Proposed mechanisms for the enhancement of T47D cell proliferation by nectin-4 and p95-ErbB2 through the Hippo signaling-dependent *SOX2* gene expression in a suspension culture. (**a**) A mechanism for nectin-4 and p95-ErbB2. Nectin-4 and p95-ErbB2 cooperatively activate not only the PI3K-AKT signaling but also the MST1/2-LATS1/2 signaling. The PI3K-AKT signaling increases the amount of the SOX2 protein by enhancing its gene expression and inhibiting its proteolytic degradation. The MST1/2-LATS1/2 signaling phosphorylates YAP, keeping the phosphorylated YAP in the cytoplasm, eventually suppressing its inhibitory role in the *SOX2* gene expression induced by the PI3K-AKT signaling. As a result, nectin-4 and p95-ErbB2 cooperatively increase the amount of the SOX2 protein, enhancing T47D cell proliferation in a suspension culture. (**b**) A mechanism for nectin-4 and either ErbB2 or ErbB2∆Ex16. Nectin-4 and either ErbB2 or ErbB2∆Ex16 activate the PI3K-AKT signaling, but not the MST1/2-LATS1/2 signaling. YAP is translocated into the nucleus and inhibits the *SOX2* gene expression. As a result, nectin-4 and either ErbB2 or ErbB2∆Ex16 do not increase the amount of the SOX2 protein.

*SOX2* gene expression is induced through at least four signaling pathways downstream of the Shh, Wnt, FGF, and TGF-β receptors^56–62^, but we showed here that none of these signaling pathways was involved in the increase in the amount of the SOX2 protein by nectin-4 and p95-ErbB2, but that the increase in the amount of the SOX2 protein by nectin-4 and p95-ErbB2 was inhibited by the MST1/2 inhibitor. MST1/2 is a signaling molecule in the Hippo signaling, which regulates diverse events, including cell proliferation and apoptosis^50^. Although YAP activation induces the *SOX2* gene expression in cancer cells, such as lung cancer^52^, we showed here that the constitutively active mutant of YAP decreased the amount of the SOX2 protein in T47D cells in an adherent culture, indicating that YAP inhibits the *SOX2* gene expression in this cell line. YAP phosphorylated by LATS1/2 is kept in the cytoplasm, suppressing its cotranscriptional activity in the nucleus^50^. Therefore, the present results, together with these previous observations^50^, indicate that MST1/2 phosphorylates and activates LATS1/2, which in turn phosphorylates YAP, keeping the phosphorylated YAP in the cytoplasm, suppressing its inhibitory role in the *SOX2* gene expression in T47D cells. Based on these lines of evidence, we propose here the following mechanisms for the increase in the amount of the SOX2 protein and the enhancement of T47D cell proliferation in a suspension culture by nectin-4 and p95-ErbB2, as schematically shown in Fig. 7a: (1) nectin-4 and p95-ErbB2 cooperatively activate not only the PI3K-AKT signaling but also the MST1/2-LATS1/2 signaling; (2) the PI3K-AKT signaling increases the amount of the SOX2 protein by enhancing its gene expression and inhibiting its proteolytic degradation; (3) the MST1/2-LATS1/2 signaling phosphorylates YAP, keeping the phosphorylated YAP in the cytoplasm, eventually suppressing its inhibitory role in the *SOX2* gene expression induced by the PI3K-AKT signaling; and (4) as a result of these processes, nectin-4 and p95-ErbB2 cooperatively increase the amount of the SOX2 protein. In contrast, nectin-4 and either ErbB2 or ErbB2∆Ex16 activate the PI3K-AKT signaling to similar extents to that by nectin-4 and p95-ErbB2 for increasing the amount of the SOX2 protein, but do not induce the MST1/2-LATS1/2 signaling and do not keep YAP in the cytoplasm, translocating YAP into the nucleus, exerting its inhibitory role in the *SOX2* gene expression, thus making nectin-4 and ErbB2 or ErbB2∆Ex16 incapable of increasing the amount of the SOX2 protein (Fig. 7b). SOX2 is a critical transcription factor that regulates cancer cell proliferation, migration, invasion, and metastasis^45–49^, and we showed here that the T47D cell proliferation in a suspension culture and the increase in the amount of the SOX2 protein were cooperatively enhanced by only the combination of nectin-4 and p95-ErbB2, but not by that of nectin-4 and either ErbB2 or ErbB2∆Ex16, and that the T47D cell proliferation in a suspension culture cooperatively enhanced by nectin-4 and p95-ErbB2 was inhibited by the MST1/2 inhibitor or *SOX2* knockdown. These results collectively indicate that only the combination of nectin-4 and p95-ErbB2, but not that of nectin-4 and either ErbB2 or ErbB2∆Ex16, cooperatively enhances T47D cell proliferation in a suspension culture through the Hippo signaling-dependent *SOX2* gene expression (Fig. 7a).

CD44, merlin, and angiomotin are upstream regulators for the Hippo signaling and associated with cell adhesion^64–68^. However, we suggested here that CD44, merlin, or angiomotin is not involved in the Hippo signaling-dependent *SOX2* gene expression and the enhancement of T47D cell proliferation by nectin-4 and p95-ErbB2 in a suspension culture. Thus, it remains elusive how only the combination of nectin-4 and p95-ErbB2, but not that of nectin-4 and either ErbB2 or ErbB2∆Ex16, cooperatively activates the Hippo signaling, because the cytoplasmic regions of ErbB2, p95-ErbB2, and ErbB2∆Ex16 are all identical^6,9,23^. One possible mechanism is that ErbB2, p95-ErbB2, and ErbB2∆Ex16, whose extracellular regions are different from each other, *cis*-interact with nectin-4 to different degrees through their extracellular regions. This is unlikely because we previously showed that ErbB2, p95-ErbB2, and ErbB2∆Ex16 *cis*-interact with nectin-4 to similar degrees^33^. The second possible mechanism is that the *cis*-interaction of necin-4 with other transmembrane proteins, such as integrin β4, affects the degrees of the *cis*-interaction of nectin-4 with ErbB2, p95-ErbB2, or ErbB2∆Ex16 through their extracellular regions, because in human breast cancer SUM190-PT cells, nectin-4 *cis*-interacts with integrin β4, eventually promoting anchorage-independent survival through the c-Src signaling^41^. The third possible mechanism is that the *trans*-interaction of necin-4 with nectin-4 or nectin-1 affects the degrees of the *cis*-interaction of nectin-4 with ErbB2, p95-ErbB2, or ErbB2∆Ex16 through their extracellular regions, because in human breast cancer SUM190-PT cells, nectin-4 not only *cis*-interacts with integrin β4 but also *trans*-interacts with nectin-1 or nectin-4 and this *tran*s-interaction further promotes anchorage-independent survival through the c-Src signaling cooperatively with integrin β4^41^. Further studies are necessary to elucidate how only the combination of nectin-4 and p95-ErbB2, but not that of nectin-4 and either ErbB2 or ErbB2∆Ex16, cooperatively activates the Hippo signaling.

Most human cancers originate from epithelial tissues, which form a sheet by cell–cell attachment through CAMs and cell–matrix attachment through integrins^55,76^. Integrins interacting extracellular matrix (ECM) molecules transduce cell survival signals mainly through c-Src and loss of integrin interactions with ECM molecules results in the initiation of a cell death program known as anchorage-independent apoptosis, anoikis^77^. However, transformed cancer cells survive in an anchorage-independent manner^50^. One mechanism for anchorage-independent cancer cell survival is that aggregation of cancer cells: tumor-derived subclones with greater metastatic capacity in vivo display increased self-aggregation in vitro and at the same time, subclones selected for increased in vitro aggregation are more metastatic in mice^78^. Invasion of the underlying stroma is frequently undertaken by large groups of tumor cells, a phenomenon known as collective, or cohort, cell migration^79,80^. Clusters of circulating tumor cells have been identified from the blood samples of breast, colorectal, prostate, and lung cancer patients as well as from mouse tumor models^81^. In human breast cancer SUM190-PT cells that express nectin-4 and nectin-1, nectin-4 *trans*-interacts with nectin-1 or nectin-4 and furthermore *cis*-interacts with integrin β4, promoting anchorage-independent survival through the SHP2-c-Src signaling in soft agar^41^. However, it remains elusive whether T47D cells proliferate in soft agar. We showed here that the T47D cells stably expressing both nectin-4 and p95-ErbB2, but not both nectin-4 and either ErbB2 or ErbB2∆Ex16, proliferated by the Hippo signaling-dependent *SOX2* gene expression in a suspension culture. It remains unknown whether circulating tumor cells proliferate in blood, but the present results raised the possibility that the T47D cells stably expressing both nectin-4 and p95-ErbB2 not only survive but also proliferate in blood.

## Methods

### Cell culture and transfection

Human breast ductal carcinoma T47D cells were purchased from ATCC (Manassas, VA, USA) and maintained in RPMI-1640 medium (D-glucose, HEPES, L-Glutamine, and phenol red included) supplemented with 10% fetal bovine serum and 10 μg/ml insulin and cultured at 37°C in 5% CO_2_. For stable expression of nectin-4 and ErbB2, stable *SOX2* knockdown using shRNA, and transient expression of YAP and its mutant in T47D cells, the plasmid of interests was introduced by electroporation using Amaxa Cell Line Nucleofector Kit V according to the manufacturer’s protocol.

### Plasmid construction

The following cDNAs were kindly provided: human *ERBB2* from Dr. T. Yamamoto (Okinawa Institute of Science and Technology Graduate University, Japan) and human *YAP* and its 5SA mutant (Serine 61 to Alanine, Serine 109 to Alanine, Serine 127 to Alanine, Serine 164 to Alanine, Serine 397 to Alanine) from Dr. H. Nishina (Tokyo Medical and Dental University, Japan). FLAG-Tagged nectin-4, GFP-tagged ErbB2, p95-ErbB2, and ErbB2ΔEx16 were constructed as described previously^33^. For expression of Myc-tagged YAP and its 5SA mutant, the cDNA fragments were amplified by PCR and inserted into pIRES-hyg3 vector (Clontech, Mountain View, CA, USA).

### Western blotting

T47D cells were cultured at 37°C for 72 h. The cells were washed with ice-cold PBS and lysed with a lysis buffer (20 mM HEPES at pH 7.5, 1% Nonidet P-40, 10% glycerol, 100 mM NaCl, 1 mM DTT, 1 mM CaCl_2_, 1.5 mM MgCl_2_, 1 mM 4-(2-aminoethyl)benzenesulfonyl fluoride hydrochloride (AEBSF), 1% sodium pyrophosphate decahydrate, protease inhibitor cocktail, and Phosphatase Inhibitor Cocktail 2 and 3). The lysates were subjected to centrifugation at 20,000×*g* for 15 min, and the supernatants were treated with an SDS sample buffer (67 mM Tris–HCl at pH 6.8, 2% SDS, 100 mM DTT, 5% sucrose, and 0.005% bromophenol blue). The samples were heated at 80°C for 2 min and subjected to SDS-PAGE followed by Western blotting. The samples separated on SDS-PAGE were transferred to polyvinylidene difluoride membranes (Merck Millipore, Billerica, MA, USA). After being blocked with Block Ace in Tris-buffered saline plus 0.05% Tween 20, the membranes were incubated with the indicated Abs using Block Ace or Can Get Signal Solution 1. After being washed three times with Tris-buffered saline plus 0.05% Tween 20, the membranes were incubated with horseradish peroxidase-conjugated IgG Abs using Block Ace or Can Get Signal Solution 2. The signals for the proteins were detected using Immobilon Western Chemiluminescent HRP Substrate.

### Abs and reagents

The Abs and reagents used in this study were listed in Supplementary Tables S1 and S2, respectively. A rat anti-E-cadherin mAb was a kind gift from Dr. M. Takeichi (Center for Biosystems and Dynamics Research, RIKEN, Japan).

### shRNA experiments

The sequences of each shRNA against human *SOX2* and a negative control shRNA were listed in Supplementary Table S3. These shRNAs were constructed in pRNAi-hU6-hyg vector (Biosettia, San Diego, CA, USA) and introduced by electroporation using Amaxa Cell Line Nucleofector Kit V according to the manufacturer’s protocol. For the selection of the T47D cells stably expressing shRNAs, hygromycin B (200 μg/ml) was used.

### Immunofluorescence microscopy

T47D cells were seeded onto coverslips and cultured for 72 h. For SOX2 immunostaining, the cells were fixed with a phosphate buffer containing 4% paraformaldehyde (Nacalai Tesque, Kyoto, Japan) at room temperature for 20 min. For E-cadherin and CD44 coimmunostaining, or E-cadherin and angiomotin coimmunostaining, the cells were fixed with Hanks’ balanced salt solution containing 10 mM HEPES (pH7.5), 2% paraformaldehyde, 1 mM CaCl_2_, 1 mM MgCl_2_, 1 mM sodium pyruvate, and 4% sucrose at 37°C for 15 min. For E-cadherin and merlin coimmunostaining, the cells were fixed with -20°C methanol for 10 min. The fixed cells were permeabilized with 0.5% Triton X-100 in PBS for 5 min. For the blocking, the permeabilized cells were treated with PBS containing 3% BSA and 5% donkey serum at room temperature for 1 h. Then, the cells were incubated with primary Abs in PBS containing 3% BSA at 4°C overnight. After three washes with PBS containing 3% BSA, the cells were incubated with secondary Abs and Hoechst33342 for nuclear staining at room temperature for 1 h. After three washes with PBS and two washes with ultra-pure water, the cells were mounted in ProLong Glass reagent. The images were acquired using a BZ-X710 microscope (KEYENCE CORPORATION, Osaka, Japan) with a CFI Plan Apo λ 4 × /0.2 and a CFI Plan Apo λ 60 × /1.4 numerical aperture objective lenses (Nikon, Inc., Tokyo, Japan) in 1920 × 1440 pixels. The displayed immunofluorescence images were applied into maximum signal intensity projection from around 40 images collected at a 0.2-μm step along the z-axis at room temperature using a software BZ-X Analyzer (KEYENCE CORPORATION, https://www.keyence.co.jp/products/microscope/fluorescence-microscope/bz-x700/models/bz-x710/). The images were processed using ImageJ version 1.48v 32-bit software (https://imagej.nih.gov/ij/) for color changes of the images. For the displayed images of anchorage-independent cell proliferation in a suspension culture, the cells were transferred to ultra-low attachment 96 well dish (IWAKI, Shizuoka, Japan). Using the 4 × /0.2 objective lens, nine images were captured to construct one-well image of 96 well dish. The captured nine images were connected as one image for each displayed image using a software BZ-X Analyzer (KEYENCE CORPORATION) with BZ-H3A Advanced Application software (KEYENCE CORPORATION, https://www.keyence.co.jp/products/microscope/fluorescence-microscope/bz-x700/models/bz-h3a/).

### Assay for cell proliferation in a suspension culture

Dish-cultured T47D cells were washed with PBS and detached with Accutase. To neutralize Accutase, an excess volume of serum-free RPMI-1640 medium was added to the detached cells. Then, the cells were collected by centrifugation, suspended in the serum-free medium, and passed through a cell strainer (30 μm, pluriSelect Life Science, Leipzig, Germany). The cells were counted and resuspended in the serum-free medium at 1.0 × 10^4^ cells/ml. The resuspended cells (1.0 × 10^3^ cells) were seeded on ultra-low attachment 6 well dish (IWAKI) with 2 ml serum-free RPMI-1640 medium supplemented with B27 supplement (vitamin A-excluded), ITS-G supplement, human EGF (20 ng/ml), human FGF (20 ng/ml), human leukemia inhibitory factor (1 μl/ml). Fresh 1 ml serum-free RPMI-1640 medium with the supplements was added every 3 days. After the culture for 28 days, the cells were collected by centrifugation and washed by HBSS containing 1 mM CaCl_2_, 1 mM MgCl_2_, 10 mM HEPES (pH7.5), and 2 mM L-alanyl-L-glutamine. The washed cells were resuspended in the HBSS and transferred to ultra-low attachment 96 well dish (IWAKI) for the image acquisition. Following the image acquisition, the cells were then re-collected and counted using DNA-amount-based cell counting kit.

### Nuclear and cytosolic fractionation

Dish-cultured T47D cells (5.0 × 10^6^ cells) were washed with PBS and detached with Accutase. To neutralize Accutase, an excess volume of serum-free RPMI-1640 medium was added to the detached cells. Then, the cells were collected by centrifugation and washed with ice-cold PBS once. The cell pellets were incubated with a hypotonic buffer (10 mM HEPES at pH 7.9, 10 mM KCl, 1.5 mM MgCl_2,_ 1 mM DTT, 1 mM AEBSF, protease inhibitor cocktail, and Phosphatase Inhibitor Cocktail 2 and 3) for 15 min on ice. After the incubation on ice, Nonidet P-40 was added to a final concentration of 0.6%. The cells were vigorously agitated and collected by centrifugation at 10,000 × g for 1 min. The supernatants were harvested and used as the cytosolic fraction. The pellets were washed with the hypotonic buffer and collected by centrifugation at 10,000×*g* for 1 min. The pellets were added an extraction buffer (20 mM HEPES at pH 7.9, 420 mM NaCl, 1.5 mM MgCl_2,_ 0.2 mM EDTA, 25% glycerol, 1 mM DTT, 1 mM AEBSF, protease inhibitor cocktail, and Phosphatase Inhibitor Cocktail 2 and 3), agitated for 15 min, and the lysates were subjected to centrifugation at 20,000×*g* for 15 min. The supernatants were harvested and used as the nuclear fraction. Each fraction was treated with the SDS sample buffer. The samples were heated at 80°C for 2 min and subjected to SDS–PAGE followed by Western blotting using the indicated Abs.

### Statistical analysis

Statistical significance was analyzed using GraphPad Prism 6 software (GraphPad Software Inc., La Jolla, CA, USA) for two-tailed Welch’s t-test for two groups, or one-way analysis of variance followed by a post hoc Tukey’s test for comparisons among more than three groups.

## Supplementary Information

Supplementary Information

## Data Availability

All the datasets analyzed and all the reagents used or generated during this study are available from the corresponding author on reasonable request.
