# Supplementary material for: Nectin-4 and p95-ErbB2 cooperatively regulate Hippo signaling-dependent SOX2 gene expression, enhancing anchorage-independent T47D cell proliferation
Source: Sci Rep. 2021 Apr 1;11:7344. doi: 10.1038/s41598-021-86437-2 (PMC8016986; doi:10.1038/s41598-021-86437-2)
Supplement: Supplementary file 1 — Supplementary Information [file 41598_2021_86437_MOESM1_ESM.pdf]

Nectin-4 and p95-ErbB2 cooperatively regulate Hippo signaling-dependent *SOX2* gene expression, enhancing anchorage-independent T47D cell proliferation

Shin Kedashiro, Takeshi Kameyama, Kiyohito Mizutani*, and Yoshimi Takai*

From the Division of Pathogenetic Signaling, Department of Biochemistry and Molecular Biology, Kobe University Graduate School of Medicine, 1-5-6 Minatojima-minamimachi, Chuo-ku, Kobe, Hyogo 650-0047, Japan

*Corresponding author:

Yoshimi Takai (ytakai@med.kobe-u.ac.jp)

or

Kiyohito Mizutani (mizutani@med.kobe-u.ac.jp)

Supplementary Figure 1: Shh, Wnt, FGF, and TGF-β receptor signaling-independent increase in the amount of the SOX2 protein by nectin-4 and p95-ErbB2 in T47D cells in an adherent culture.

Supplementary Figure 2: CD44, angiomotin, or merlin-independent costimulatory effects of nectin-4 and p95-ErbB2 in T47D cells in an adherent culture.

Supplementary Figure 3: Full-length blots of Figure 1c.

Supplementary Figure 4: Full-length blots of Figure 3c and f.

Supplementary Figure 5: Full-length blots of Figure 4c.

Supplementary Figure 6: Full-length blots of Figure 5a–c.

Supplementary Figure 7: Full-length blots of Supplementary Figure 1.

Supplementary Figure 8: Full-length blots of Supplementary Figure 2d.

Supplementary Table 1: The list of the antibodies used in this study.

Supplementary Table 2: The list of the reagents used in this study.

Supplementary Table 3: The list of the shRNA sequences used in this study.

Supplementary Fig 1

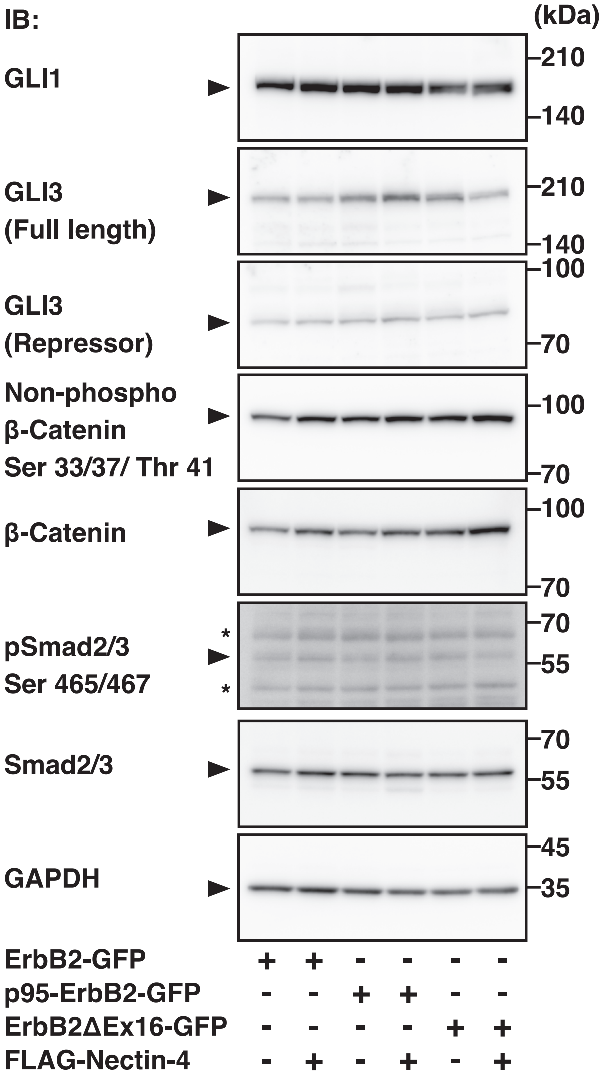

Supplementary Figure 1. Shh, Wnt, FGF, and TGF-β receptor signaling-independent increase in the amount of the SOX2 protein by nectin-4 and p95-ErbB2 in T47D cells in an adherent culture. The T47D cells stably expressing GFP-tagged ErbB2 or each of its splice variants with FLAG-tagged nectin-4 (FLAG-Nectin-4) were cultured for 72 hours in an adherent culture. The cells were subjected to Western blotting using the indicated Abs. All the cell lines used in the experiments were the bulk of collected cells and not singly picked-up clones. Arrowheads and square brackets indicate each of the proteins. Asterisks indicate non-specific bands. The displayed blots were cropped, and the full-length blots are shown in **Supplementary Figure 7**. IB, immunoblotting. Representative results from three independent experiments are shown.

Supplementary Fig 2

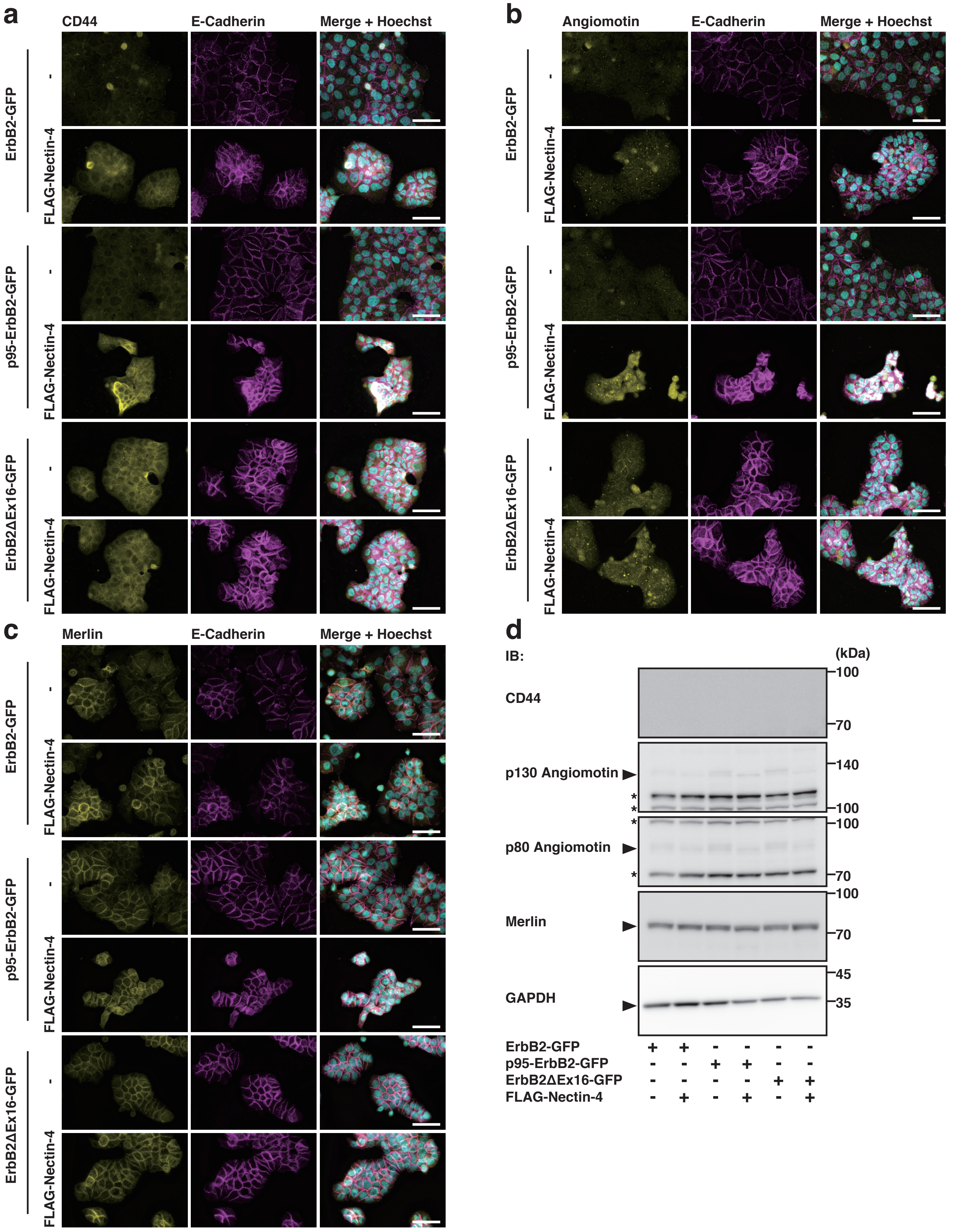

Supplementary Figure 2. CD44, angiomotin, or merlin-independent costimulatory effects of nectin-4 and p95-ErbB2 in T47D cells in an adherent culture. a–d CD44, angiomotin, or merlin-independent costimulatory effects of nectin-4 and p95-ErbB2 in T47D cells cultured in an adherent culture. The T47D cells stably expressing GFP-tagged ErbB2 or each of its splice variants with FLAG-tagged nectin-4 (FLAG-Nectin-4) were cultured for 72 hours on dishes. The cells were fixed and stained with the indicated antibody and Hoechst33342, or subjected to Western blotting using the indicated Abs as shown in (**a–d**). The displayed images were acquired using a BZ-X710 microscope and its software BZ-X Analyzer (https://www.keyence.co.jp/products/microscope/fluorescence-microscope/bz-x700/models/bz-x710/) and processed using ImageJ version 1.48v 32-bit software (https://imagej.nih.gov/ij/) for color changes of the images. All the cell lines used in the experiments were the bulk of collected cells and not singly picked-up clones. Arrowheads and square brackets indicate each of the proteins. Asterisks indicate non-specific bands. The displayed blots were cropped, and the full-length blots are shown in **Supplementary Figure 8**. Scale bars, 50 μm. IB, immunoblotting. Representative results (images) from three independent experiments were shown.

Supplementary Fig 3

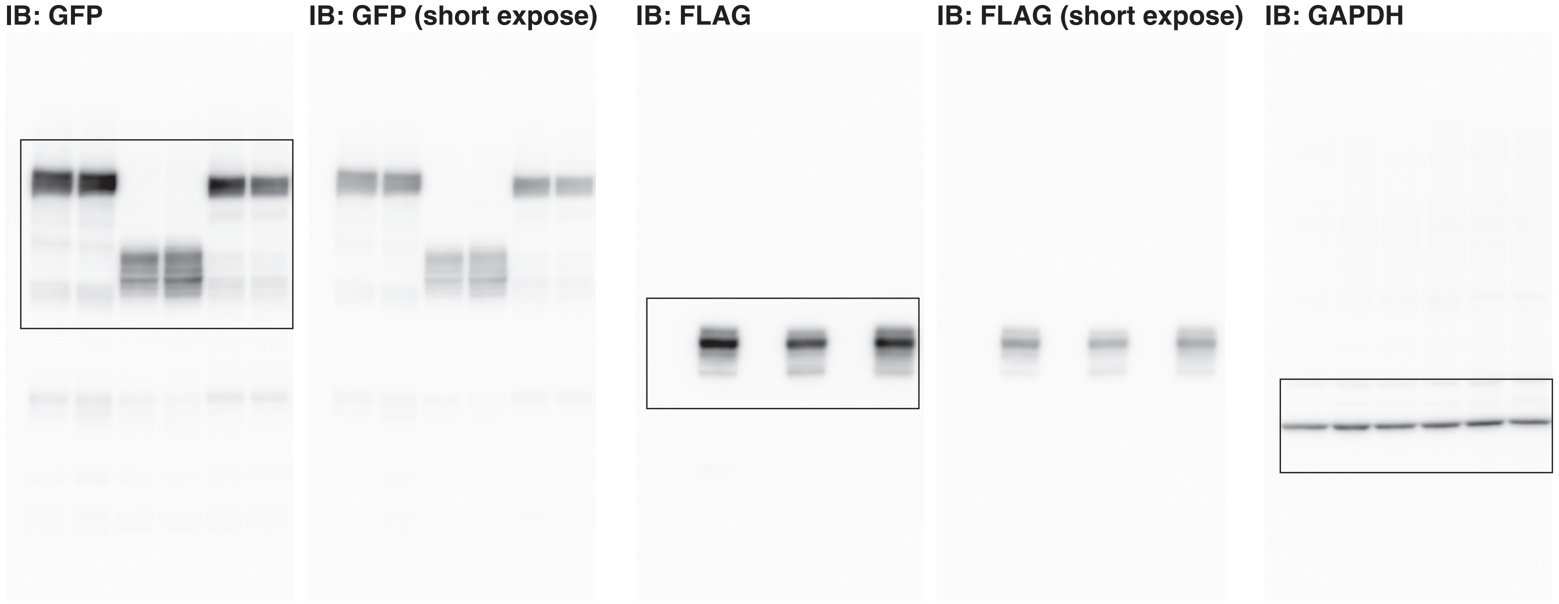

Supplementary Figure 3. Full-length blots of Figure 1c. Full-length blots of Figure 1c in this manuscript. Boxed regions were cropped and shown as the figure. IB, immunoblotting.

Supplementary Fig 4

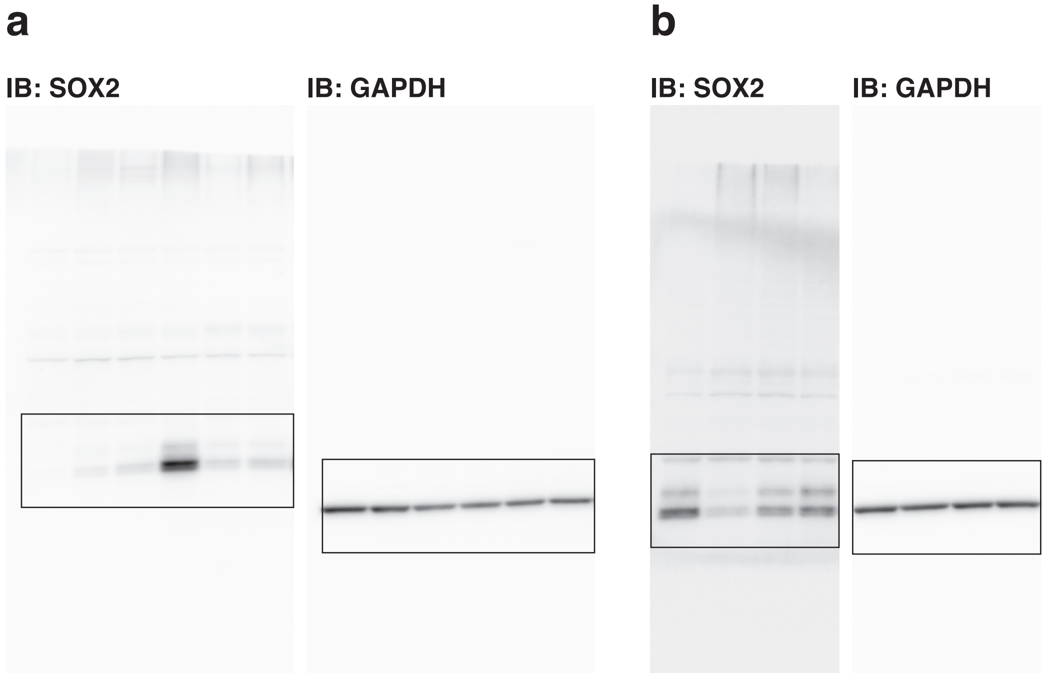

Supplementary Figure 4. Full-length blots of Figure 3c and f. a, b Full-length blots of Figure 3c and f, respectively. Boxed regions were cropped and shown as the figure. IB, immunoblotting.

Supplementary Fig 5

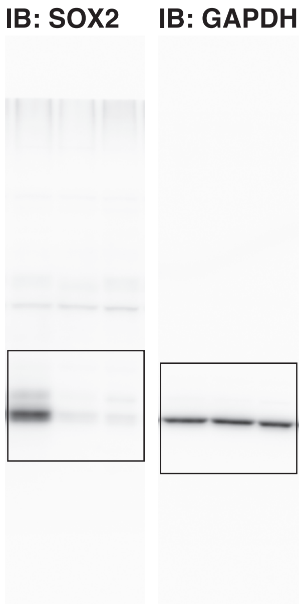

Supplementary Figure 5. Full-length blots of Figure 4c. Full-length blots of Figure 4c in this manuscript. Boxed regions were cropped and shown as the figure. IB, immunoblotting.

Supplementary Fig 6

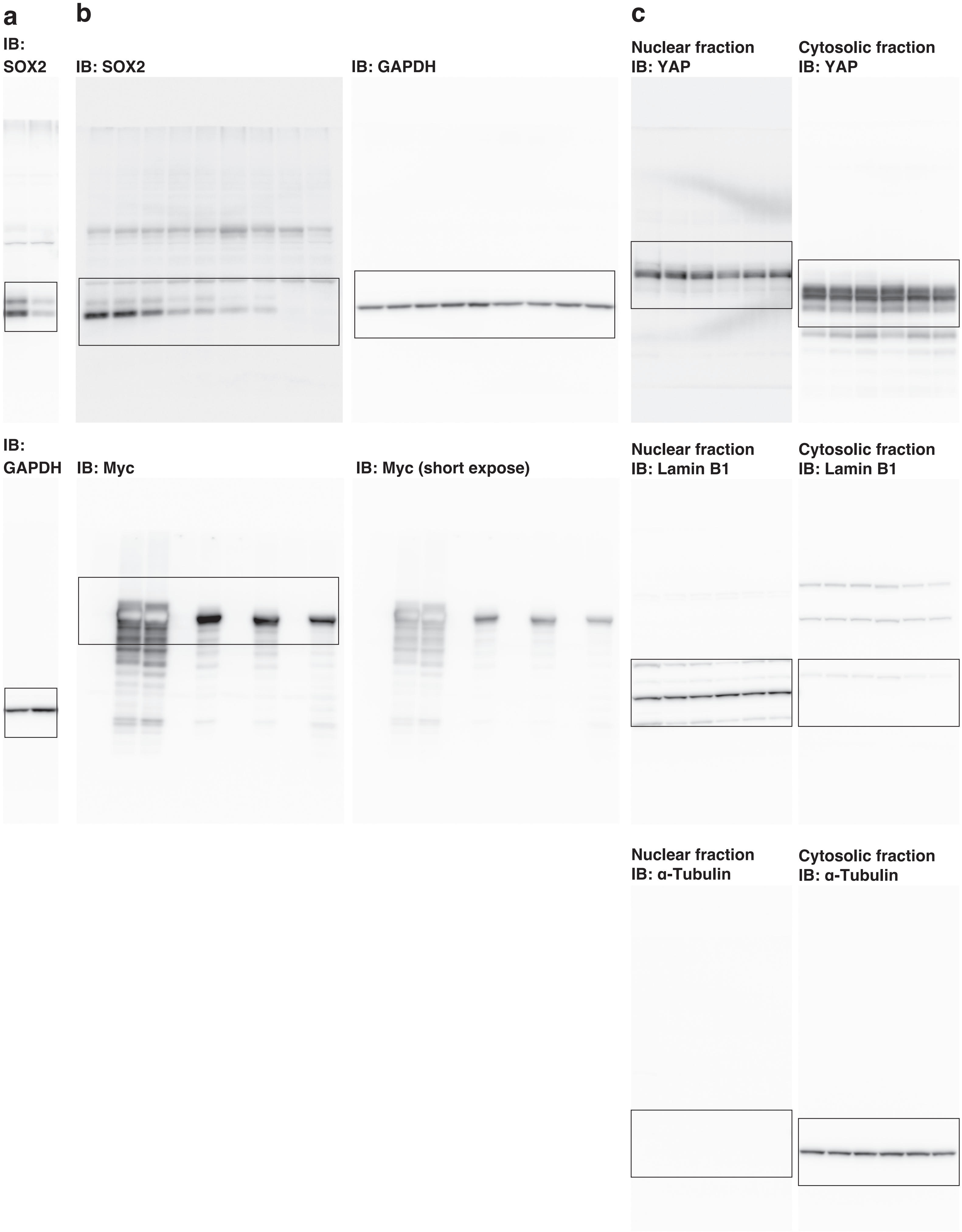

Supplementary Figure 6. Full-length blots of Figure 5a–c. a–c Full-length blots of Figure 5a–c, respectively. Boxed regions were cropped and shown as the figure. IB, immunoblotting.

Supplementary Fig 7

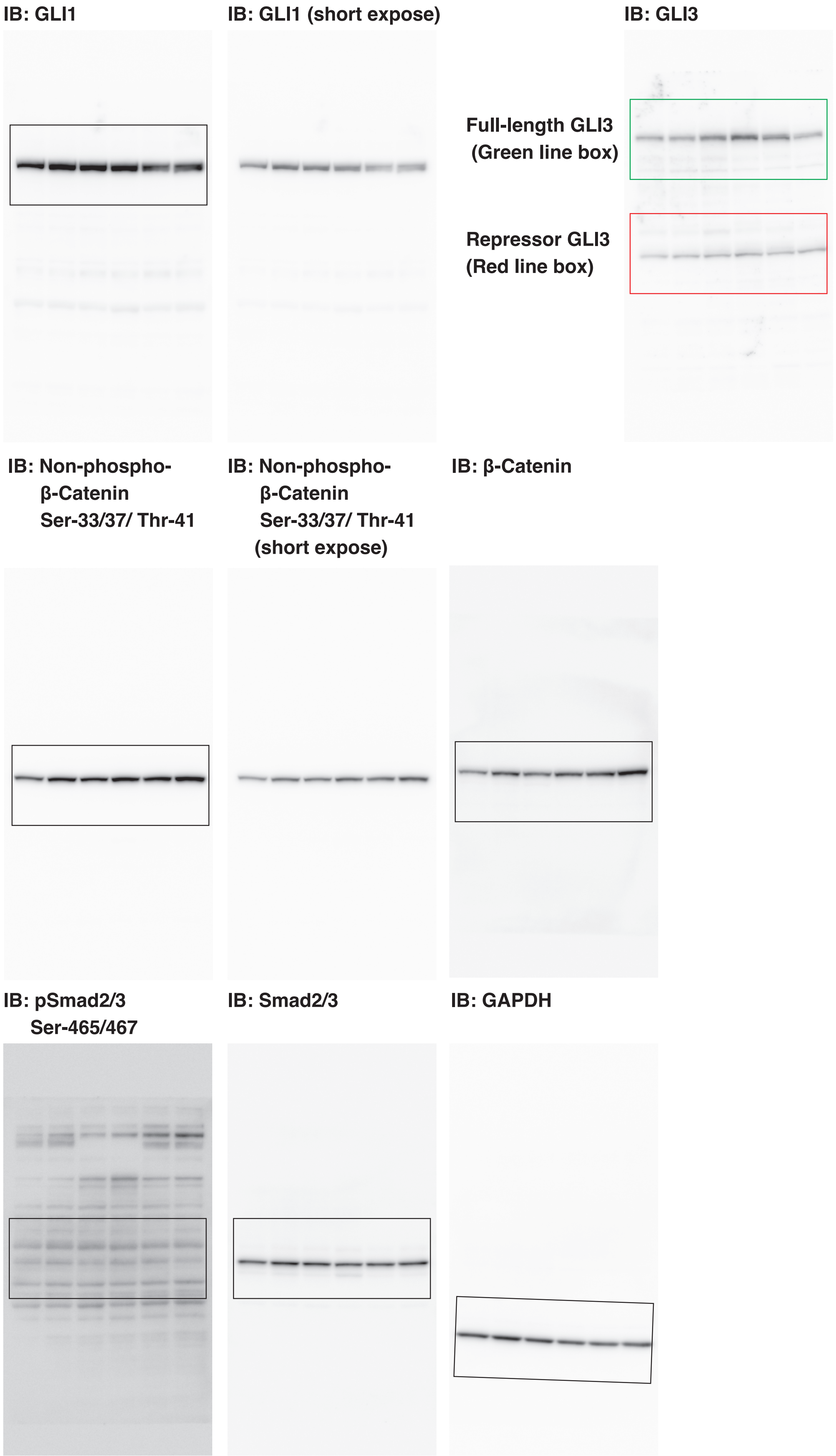

Supplementary Figure 7. Full-length blots of Supplementary Figure 1. Full-length blots of Supplementary Figure 1 in this manuscript. Immunoblots of full-length GLI3 and repressor GLI3 were obtained from the same membrane. Boxed regions were cropped and shown as the figure. Green line box, full-length GLI3; and red line box, repressor GLI3. IB, immunoblotting.

Supplementary Fig 8

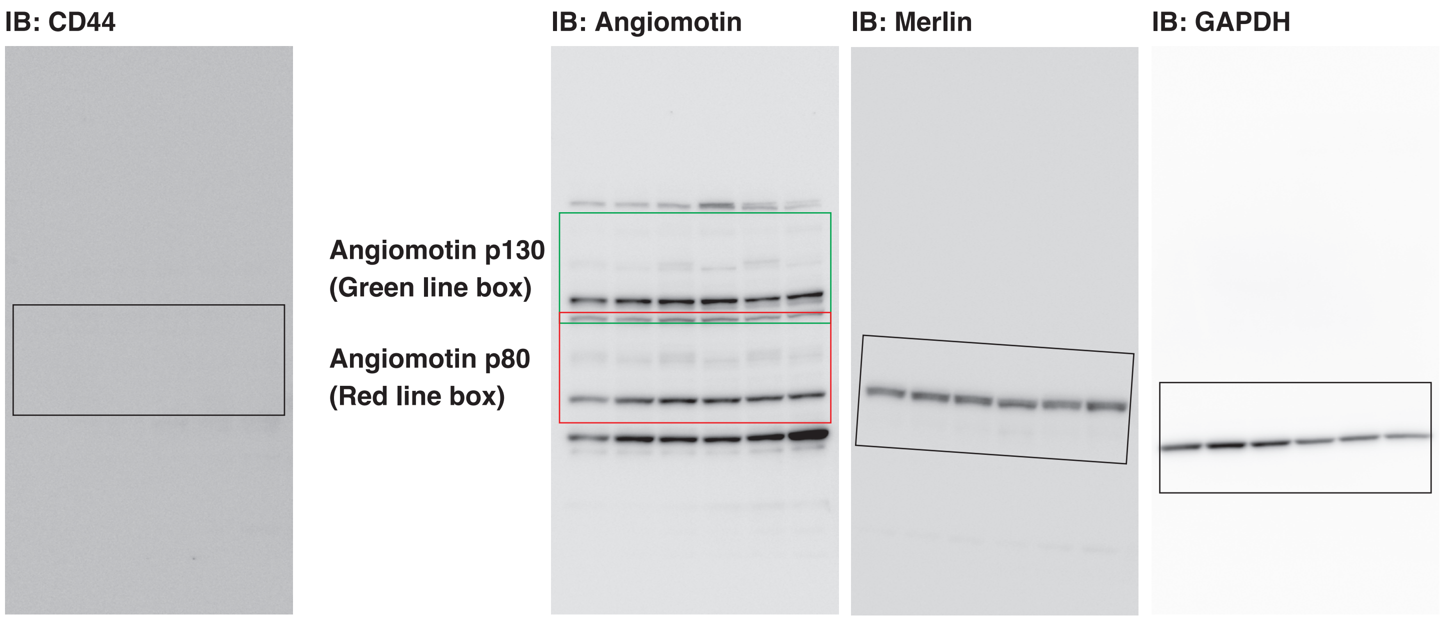

Supplementary Figure 8. Full-length blots of Supplementary Figure 2d. Full-length blots of Supplementary Figure 2d in this manuscript. Immunoblots of angiomotin p130 and angiomotin p80 were obtained from the same membrane. Boxed regions were cropped and shown as the figure. Green line box, angiomotin p130; and red line box, angiomotin p80. IB, immunoblotting.

Supplementary Table 1

Antibody	Use	Dilution	Buffer	Host	Clone	Clone #	Supplier	Address	Catalogue #
**Primary antibody**									
Angiomotin	IB	1 / 500	Block Ace	Rb	Poly	-	PROTEINTECH	Rosemont, IL, USA	24550-1-AP
β-Catenin	IB	1 / 1000	Block Ace	Rb	Mono	D10A8	Cell Signaling Technology	Danvers, MA, USA	8480
Non-phospho-β-Catenin (Ser33/37/Thr41)	IB	1 / 1000	Block Ace	Rb	Mono	D13A1	Cell Signaling Technology	Danvers, MA, USA	8814
CD44	IB, IF	IB: 1 / 1000, IF: 1 / 100	IB: Block Ace, IF: PBS with 3% BSA	Rb	Mono	E7K2Y	Cell Signaling Technology	Danvers, MA, USA	37259
E-Cadherin	IF	1 / 500	PBS with 3% BSA	Rt	Mono	ECCD2	-	-	Reffered in Methods
FLAG	IB	1 / 1000	Block Ace	Ms	Mono	M2	Sigma-Aldrich	St. Louis, MO, USA	F3165
GAPDH	IB	1 / 1000	Block Ace	Rb	Mono	14C10	Cell Signaling Technology	Danvers, MA, USA	2118
GFP	IB	1 / 1000	Block Ace	Rb	Poly	-	MBL International	Nagoya, Japan	598
GLI1	IB	1 / 1000	Block Ace	Ms	Mono	1D2B2	PROTEINTECH	Rosemont, IL, USA	66905-1-Ig
GLI3	IB	1 / 500	Block Ace	Gt	Poly	-	R&D Systems, Inc.	Minneapolis, MN, USA	AF3690
Lamin B1	IB	1 / 1000	Block Ace	Rb	Poly	-	PROTEINTECH	Rosemont, IL, USA	12987-1-AP
Merlin	IB, IF	IB: 1 / 1000, IF: 1 / 100	IB: Block Ace, IF: PBS with 3% BSA	Rb	Mono	D3S3W	Cell Signaling Technology	Danvers, MA, USA	12888
Myc-tag	IB	1 / 1000	Block Ace	Rb	Poly	-	MBL International	Nagoya, Japan	562
Smad2/3	IB	1 / 1000	Block Ace	Rb	Mono	D7G7	Cell Signaling Technology	Danvers, MA, USA	8685
Phospho-Smad2 (Ser465/467)/Smad3 (Ser423/425)	IB	1 / 5000	Can Get	Rb	Mono	D27F4	Cell Signaling Technology	Danvers, MA, USA	8828
SOX2	IB	1 / 1000	Block Ace	Rb	Poly	-	PROTEINTECH	Rosemont, IL, USA	20118-1-AP
SOX2 (eFluor 570 conjugate)	IF	1 / 200	PBS with 3% BSA	Rt	Mono	Btjce	Thermo Fisher Scientific	Waltham, MA, USA	41-9811-82
α-Tubulin	IB	1 / 2000	Block Ace	Ms	Mono	B-5-1-2	Sigma-Aldrich	St. Louis, MO, USA	T5168
YAP	IB	1 / 1000	Block Ace	Rb	Mono	D8H1X	Cell Signaling Technology	Danvers, MA, USA	14047
**Secondaty antibody**									
Mouse IgG-HRP	IB	1 / 10000	Block Ace	Sheep	Poly	-	GE Healthcare	Little Chalfont, UK	NA931
Rabbit IgG-HRP	IB	1 / 10000	Block Ace or Can Get	Donk	Poly	-	GE Healthcare	Little Chalfont, UK	NA934
Goat IgG-HRP	IB	1 / 10000	Block Ace	Rb	Poly	-	MBL International	Nagoya, Japan	546
Rabbit IgG-Alexa Fluor 555	IF	1 / 200	PBS with 3% BSA	Donk	Poly	-	Abcam	Cambridge, UK	ab150074
Rat IgG-Alexa Fluor 647	IF	1 / 200	PBS with 3% BSA	Donk	Poly	-	Abcam	Cambridge, UK	ab150155

IB, immunoblotting; IF, immunofluorescence; Can Get, Can Get Signal Solution; Rb, Rabbit; Rt, Rat; Ms, Mouse; Gt, Goat; Donk, Donkey; Poly, Polyclonal; Mono, Monoclonal; HRP, horseradish peroxidase.

Supplementary Table 2

Reagent	Use	Supplier	Address	Catalogue #
**Cell culture and transfection**				
Accutase	Detachment for cell	Nacalai Tesque	Kyoto, Japan	12679-54
Amaxa Cell Line Nucleofector Kit V	Transfection	Lonza	Allendale, NJ, USA	VCA-1003
B27 Supplement, minus vitamin A	Supplement for cell culture	Thermo Fisher Scientific	Waltham, MA, USA	12587010
DNA-amount-based cell counting kit (Cyquant assay)	Counting cell number	Thermo Fisher Scientific	Waltham, MA, USA	C7026
Epidermal growth factor	Supplement for cell culture	FUJIFILM Wako Pure Chemical Corporation	Osaka, Japan	059-07873
Fibroblast growth factor (basic)	Supplement for cell culture	FUJIFILM Wako Pure Chemical Corporation	Osaka, Japan	064-05381
HEPES	Supplement for Hank's balanced salt solution	Nacalai Tesque	Kyoto, Japan	15639-84
Hygromycin B	Cell selection for stably expressing shRNAs	Nacalai Tesque	Kyoto, Japan	09287-84
Insulin	Supplement for cell culture	FUJIFILM Wako Pure Chemical Corporation	Osaka, Japan	093-06351
ITS-G supplement	Supplement for cell culture	FUJIFILM Wako Pure Chemical Corporation	Osaka, Japan	090-06741
L-alanyl-L-glutamine	Supplement for Hank's balanced salt solution	Nacalai Tesque	Kyoto, Japan	04260-64
Leukemia inhibitory factor	Supplement for cell culture	FUJIFILM Wako Pure Chemical Corporation	Osaka, Japan	129-05601
LY294002	Inhibitor for cell culture	FUJIFILM Wako Pure Chemical Corporation	Osaka, Japan	129-04861
RPMI-1640	Medium for cell culture	FUJIFILM Wako Pure Chemical Corporation	Osaka, Japan	189-02145
Ruxolitinib	Inhibitor for cell culture	Selleck Chemicals	Houston, TX, USA	S1378
U0126	Inhibitor for cell culture	Calbiochem	Billerica, MA, USA	662005
XMU-MP-1	Inhibitor for cell culture	Selleck Chemicals	Houston, TX, USA	S8334
**Cell staining and WB**				
Block Ace	IB	KAC	Kyoto, Japan	UKB80
Can Get Signal Solution 1	IB	Toyobo	Osaka, Japan	NKB-201
Can Get Signal Solution 2	IB	Toyobo	Osaka, Japan	NKB-301
Donkey serum	Blocking for cell staining	Merck Millipore	Billerica, MA, USA	S30-100ML
Hoechst33342	Cell staining	FUJIFILM Wako Pure Chemical Corporation	Osaka, Japan	12679-54
Immobilon Western Chemiluminescent HRP Substrate	IB	Merck Millipore	Billerica, MA, USA	WBKLS0500
Phosphatase Inhibitor Cocktail 2	IB	Sigma-Aldrich	St. Louis, MO, USA	P5726
Phosphatase Inhibitor Cocktail 3	IB	Sigma-Aldrich	St. Louis, MO, USA	P0044
ProLong Glass reagent	Cell staining	Thermo Fisher Scientific	Waltham, MA, USA	P36980
Protease inhibitor cocktail (cOmplete, EDTA-free)	IB	Roche Diagnostics GmbH	Mannheim, Germany	11836170001

IB, immunoblotting; HRP, horseradish peroxidase.

Supplementary Table 3

**shRNA**	**Taget sequence**
Control	5'-CAACAAGATGAAGAGCACCAA-3'
Human *SOX2* #1	5'-CGAGATAAACATGGCAATCAA-3'
Human *SOX2* #2	5'-GGAGCACCCGGATTATAAA-3'
